# A Conditionally Activated Cytosol-Penetrating Antibody for TME-Dependent Intracellular Cargo Delivery

**DOI:** 10.3390/antib13020037

**Published:** 2024-05-02

**Authors:** Carolin Sophie Dombrowsky, Dominic Happel, Jan Habermann, Sarah Hofmann, Sasi Otmi, Benny Cohen, Harald Kolmar

**Affiliations:** 1 Institute for Organic Chemistry and Biochemistry, Technical University of Darmstadt, Peter-Grünberg-Strasse 4, D-64287 Darmstadt, Germany; 2 Inter-Lab, a Subsidiary of Merck KGaA, South Industrial Area, Yavne 8122004, Israel; 3 Centre for Synthetic Biology, Technical University of Darmstadt, D-64287 Darmstadt, Germany

**Keywords:** cytosol-penetrating antibody, cytosolic delivery, endosomal escape, masked cytosol-penetrating antibody, MMP-9, TME

## Abstract

Currently, therapeutic and diagnostic applications of antibodies are primarily limited to cell surface-exposed and extracellular proteins. However, research has been conducted on cell-penetrating peptides (CPP), as well as cytosol-penetrating antibodies, to overcome these limitations. In this context, a heparin sulfate proteoglycan (HSPG)-binding antibody was serendipitously discovered, which eventually localizes to the cytosol of target cells. Functional characterization revealed that the tested antibody has beneficial cytosol-penetrating capabilities and can deliver cargo proteins (up to 70 kDa) to the cytosol. To achieve tumor-specific cell targeting and cargo delivery through conditional activation of the cell-penetrating antibody in the tumor microenvironment, a single-chain Fc fragment (scFv) and a V_L_ domain were isolated as masking units. Several in vitro assays demonstrated that fusing the masking protein with a cleavable linker to the cell penetration antibody results in the inactivation of antibody cell binding and internalization. Removal of the mask via MMP-9 protease cleavage, a protease that is frequently overexpressed in the tumor microenvironment (TME), led to complete regeneration of binding and cytosol-penetrating capabilities. Masked and conditionally activated cytosol-penetrating antibodies have the potential to serve as a modular platform for delivering protein cargoes addressing intracellular targets in tumor cells.

## 1. Introduction

Nowadays, antibodies in various formats are used as therapy for autoimmune diseases, viral infections, and various types of cancer [1,2,3]. Due to their size of approximately 150 kDa, they typically target receptors on the cell surface or extracellular proteins. Consequently, numerous disease-related protein–protein interactions that occur in the cytosol cannot be targeted without cell lysis or permeation [4,5]. Intracellular delivery requires overcoming the barrier of the cell membrane. Over the years, various pathways have been utilized for cargo internalization. This can occur either through direct cell membrane penetration, which mainly applies to hydrophobic small molecules, or through endocytosis, in the case of macromolecules [6,7]. The latter pathway mainly results in either recycling of the protein to the cell surface or proteolysis in lysosomes [7]. Efficient endosomal uptake of antibodies, receptor binding, and subsequent release in the endosome are necessary for the cytosolic delivery. Eventually, the antibodies have to pass through the endosomal membrane.

In the past, it has been observed that some antibodies associated with the autoimmune disease Systemic Lupus Erythematosus (SLE) bind to double-stranded DNA (anti-dsDNA antibodies) and can penetrate the cytosol [8,9,10,11]. Further studies have shown that cellular uptake is initiated by binding to heparan sulfate proteoglycan (HSPG), a glycoprotein that is found on the surface of most cell types [8,12]. HSPGs are covalently attached to one or more heparan sulfate (HS) side chains [13]. These chains perform multiple functions in and on cells, including involvement in cell–cell interactions, cytoskeletal interactions, and transcellular transport [13]. The diverse structure and composition of HS side chains contribute to their versatility [13]. The overexpression of the HS cleaving endoglycosidase heparanase (HSPE) in several human tumors plays a crucial role in the release and distribution of growth factors, cytokines, and the remodeling of the extracellular matrix. HSPE is a pivotal factor in tumor progression and metastasis [14,15]. Various HS binding sequence motifs in the CDRs of HSPG-binding antibodies were identified, including XBBXBX, XBBBXXBX, and XBBBXXBBBXXBBX, with ‘B’ representing a basic amino acid and ‘X’ representing a random amino acid residue [16,17,18]. These motifs bind to the negatively charged side chains of HSPG and dsDNA, due to an increased number of positively charged amino acids [16,17,18]. The proteoglycan–antibody complex is internalized through receptor-mediated endocytosis as a result of the electrostatic interaction between the binding motif and HSPG. Proteolytic processing in the endosome modifies internalized HSPE, leading to dissociation of the antibody from the HSPG and release of the antibody in the endosome [19,20,21]. The mechanism by which the antibody enters the cell cytoplasm upon endosomal escape is not yet fully understood. TMab4 cytotransmab [22] is one of the few well-characterized internalizing antibodies and contains a HSPG-binding motif in the CDR3 of the light chain [22]. Mutation studies suggest that TMab4 cytotransmab localizes in the cytosol through endosomal escape motifs YYH (TyrL92, TyrL93, and HisL94) or WYW (TrpL92, TyrL93, and TrpL94) located in CDR-L3 [23,24,25]. The decrease in pH within the endosomes induces local conformational changes in the antibody loop, promoting pore formation in the endosomal membrane through the hydrophobic aromatic triple residue motif, ultimately leading to the release of the antibody into the cytosol [23]. Notably, HSPG is present on the surface of most cell types, making cytosol penetration unspecific [12].

In recent years, antibody tumor therapy has not only focused on direct interference with tumor cell surface receptors but has also considered the special characteristics of the tumor microenvironment (TME). The TME can be defined as the immediate environment of a malignant tumor in an organism with a high variation of composition between different tumor types [26]. Besides cancerous tumor cells, the highly heterogeneous TME, including immune cells, blood vessels, stromal cells, and extracellular matrix, has a significant effect on tumor growth and progression [26,27]. Several proteases, such as MMP-2, MMP-9, or matriptase, have been described to accumulate in the tumor microenvironment [28]. These tissue-dependent proteases are naturally involved in the degradation of the extracellular matrix and can promote tumor progression and the formation of metastases [28,29,30,31].

For tumor-specific targeting by antibodies and reduction of off-target binding, several masking strategies have been developed. These strategies often involve placing a masking unit at the antigen-binding site of a tumor-targeting therapeutic antibody. The two modules are linked through a cleavable linker, which allows for proteolytic demasking in the TME and restoration of tumor antigen binding. Various masking units have been utilized to inhibit the binding capability of the antibody, either through steric hindrance (such as coiled-coils [32]) or a paratope-specific interaction [33,34]. The use of an anti-idiotypic single chain Fv (scFv) fragment as a masking entity, which binds to key CDRs for target binding, can achieve nearly complete blocking of antigen binding [35].

**Figure 1:**
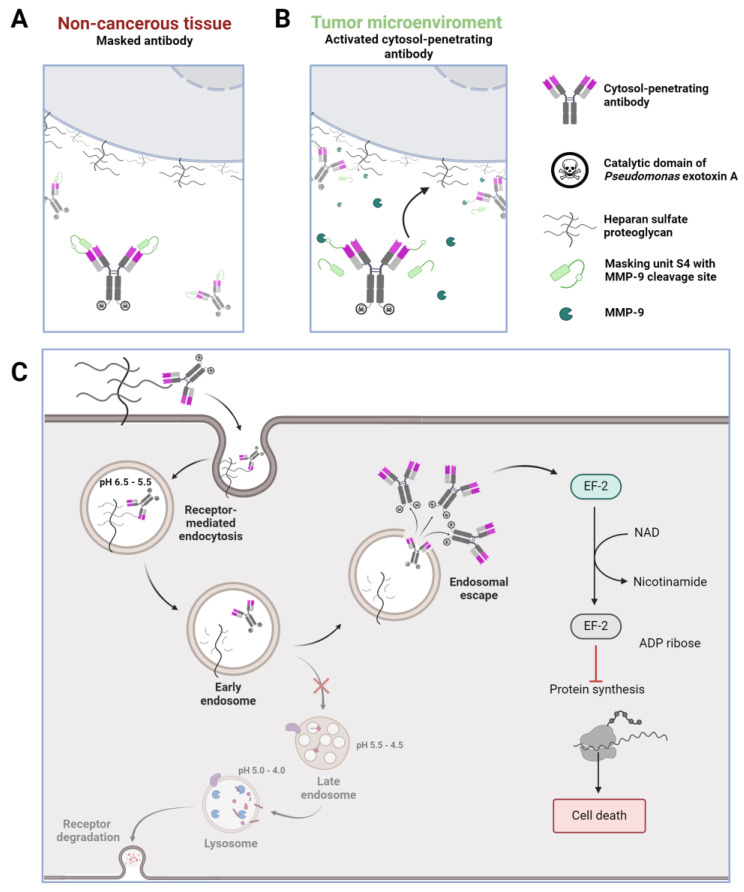
Schematic overview of the (**A**) masking capabilities in non-cancerous tissue, in comparison to (**B**) activation and restoration of binding capabilities after TME-associated MMP-9 cleavage, and (**C**) the pathway of the activated cytosol-penetrating antibody after HSPG binding on the cell surface. The antibody is released from HSPG through receptor-mediated endocytosis, followed by internalization and subsequent release. Decrease in pH promotes endosomal escape of the cytosol-penetrating antibody with attached cargo, which is, in this instance, C-terminally truncated *P. aeruginosa* exotoxin. This figure was created using BioRender.com.

This study utilized the masking concept to generate a conditionally activated HSPG-binding and cell-permeating antibody, named CPAb (cytosol-penetrating antibody). We compared its general cell internalization and cytosol-penetrating capabilities to the previously published and well-characterized HerT4 cytotransmab [22]. The application of two orthogonal cell assays confirmed the cytosolic localization of the CPAb with enhanced cytosolic penetration, compared to the HerT4 cytotransmab. Additionally, two assays with CPAb validated the cytosolic delivery of cargo proteins, with a total molecular weight of up to 70 kDa using eGFP and truncated *Pseudomonas aeruginosa* exotoxin. Finally, a masked cytosol-penetrating antibody was generated that showed significantly reduced cytosolic penetration that was re-established upon MMP-9 cleavage of the mask, as illustrated in Figure 1.

To the best of our knowledge we demonstrate, for the first time, that masking of a cell-penetrating antibody through fusion of a masking unit with a protease-cleavable linker results in conditional activation of internalization and cytoplasmic location via cleavage of a protease present in the tumor microenvironment.

## 2. Materials and Methods

### 2.1. Plasmids

The pSF3_LgBiT plasmid was constructed by usage of two gene strings consisting of either LgBiT or stop codon sequences (Twist Bioscience, South San Francisco, CA, USA) as templates for restriction with *EcoRI/NotI* and *HindIII/BamHI*, respectively. As a template for insertion, pSF3_microID_NES plasmid was used, having been provided by Julien Béthune (Department of Biotechnology, Hamburg University of Applied Sciences) [36].

The plasmid for bacterial production of the truncated catalytic domain of *Pseudomonas* exotoxin (PE_cat_; sequence in Table S1) was constructed from the pMAL_MBP-Furin-Gelonin-LPETGS containing maltose-binding protein (previously described by Thomas Pirzer et al. [37]) and a gene string (Twist Bioscience) with (G_4_S)_2_-PE_cat_, where the C-terminal translocation sequence (KDEL; [38]) was deleted and replaced by a dual purification tag including His_6_-tag and Twin-Strep-tag^®^ (WSHPQFEKGGGSGGGSGGSSAWSHPQFEK; [39]) for two-step affinity purification.

For the generation of the pET30_(G4S)-eGFP plasmid for sortase A coupling, a DNA sequence of eGFP (UniProtKB: C5MKY7) was utilized as template for amplification of N-terminal insertion of a His_6_-purification tag and a (G_4_S)-linker separated by TEV recognition site (ENLYFQG).

Protocols, cell media, plasmids, primers, and cell strains for recombinant expression and purification of solitaire scFvs were previously described. Plasmid and DNA preparations for yeast surface display of chicken-derived scFvs, as well as handling of yeast cells, were extensively detailed earlier [40,41].

The pTT5-hT4-LC plasmid (Invitrogen, Waltham, MA, USA) was designed on the basis of the origin of Choi, Bae et al. [22].

The parental pTT5-CPAb-HC and pTT5-CPAb-LC plasmids were provided by Benny Cohen (Inter-Lab, a subsidiary of Merck KGaA, South Industrial Area, Yavne 8122004, Israel). The mutated CPAb (118S–121S) variant was constructed by restriction-free cloning. For the integration of the sortase A LPETGG tag to the C-terminus of the trastuzumab, CPAb or CPAb (118S–121S) heavy chain, the reverse primer 5′-TATATATAGCTCTTCTTCCTTAACCACCGGTTTCCGGCAGTGAACCGCCCCCGCCTTTACCCGGGGACAGGGAGAG-3′ was used; and for the addition of the HiBiT peptide, the reverse primer 5′-TATATATATAATATGCTCTTCATCCTCAGCTAATCTTCTGAACAGCCGCCAGCCGCTCACATTCCACCACCACCTTTACCCGG-3′ was used. The correct plasmid sequences were verified by sequencing (Microsynth Seqlab GmbH, Göttingen, Germany).

### 2.2. Cell Lines

A-431, HeLa, and SKBR-3 cells were cultivated in Dulbecco’s Modified Eagle Medium high glucose (Thermo Fisher Scientific, Waltham, MA, USA) supplemented with 10% FBS (Sigma–Aldrich, St. Louis, MO, USA) and 1% Penicillin-Streptomycin (Thermo Fisher Scientific) at 37 °C and 5% CO_2_. The cells were sub-cultured every 3–4 days. The Expi293F™ HEK cells were cultured in Expi293™ Expression Medium (Thermo Fisher Scientific) at 37 °C, 8% CO_2_ and 110 rpm. Sub-culturing was carried out every 3–4 days.

The HeLa 11ht_LgBiT cell line was generated by transfection of HeLa EM2-11ht (provided by Julien Béthune, Department of Biotechnology, Hamburg University of Applied Sciences) with pSF3_LgBiT and pCAGGS-FLPe-IRESpuro (Addgene: 13793; Watertown, MA, USA), as previously described in [36] or originally by Weidenfeld et al. in [42]. Generated single clones were verified by transient transfection with pTT5_CPAb-HC_HiBiT, and following NanoBiT^®^ luciferase assay. The stably transfected HeLa 11ht_LgBiT cells were cultivated in Dulbecco’s Modified Eagle Medium high glucose (Thermo Fisher Scientific) supplemented with 10% FBS (Sigma–Aldrich), 1% Penicillin-Streptomycin (Thermo Fisher Scientific), 0.2 mg/mL G418 and 40 µM ganciclovir at 37 °C and 5% CO_2,_ and sub-cultured every 3–4 days.

### 2.3. Expression and Purification of Full-Length Antibodies and PE Toxin

The expression of full-length antibodies was conducted with pTT5 vectors in Expi293F™ HEK cells (Thermo Fisher Scientific). The transient transfection of Expi293F™ HEK cells were either performed using ExpiFectamine™ 293 Transfection Kit (Thermo Fisher Scientific) or PEI-Transferrinfection Kit (Invitrogen), following the manufacturer’s protocol. Five days post-transfection, the cell supernatant was sterile filtered and applied to a HiTrap™ Protein A HP column (GE Healthcare, Chicago, IL, USA) using an ÄKTA start™ chromatography system (GE Healthcare). The buffer was exchanged to either PBS or SrtA buffer (0.05 M Tris, 0.15 M NaCl, 0.005 M CaCl_2_, pH 7.5), depending on the following applications.

The (G_4_S)_2_-PE_cat_ was expressed as fusion protein with maltose-binding protein (MBP) in a pMALC vector in *E. coli* BL21 (DE3) cells. The electrocompetent *E. coli* BL21 (DE3) cells were transformed with pET30_MBP-PE_cat_-His-Strep and cultivated in dYT supplemented with ampicillin at 37 °C and 180 rpm. One day post-transformation, 1 L dYT was inoculated with transformed pre-culture and was grown to an OD_600nm_ of 0.6–0.8. The protein expression was induced by 0.5 mL 1 M IPTG, followed by the incubation at 25 °C and 180 rpm overnight. The purification of the fusion protein was performed via two-step affinity purification utilizing IMAC and Strep–Tactin^®^XT columns. For this, the *E. coli* pellet was resuspended in running buffer (0.05 M TRIS, 0.15 M NaCl, 0.02 M Imidazole; pH 7.5), incubated at −80 °C for 30 min and lysed afterwards using an ultrasonic electrode. The supernatant was sterile filtered and applied to the HisTrap™ HP column (1 mL, GE Healthcare) and Strep–Tactin^®^XT 4Flow column (1 mL, IBA Lifesciences, Göttingen, Germany) using an ÄKTA start™ chromatography system (GE Healthcare). The buffer was exchanged to PBS by dialysis overnight.

The (G_4_S)-eGFP was expressed in *E. coli* BL21 (DE3) cells after electroporation of pET30_e (G4S)-GFP. One day after transformation, 1 L TB medium (24 g/L yeast extract, 20 g/L tryptone and 4 mL/L glycerol) was inoculated with the pre-culture, grown to an OD_600nm_ of 0.6–0.8, and protein expression was induced by supplementation with 0.5 mL 1 M IPTG. The culture was incubated at 25 °C and 180 rpm overnight. Protein purification was performed via IMAC purification, as previously described. The buffer was exchanged to PBS by dialysis overnight.

### 2.4. Protein Conjugation Reactions

The coupling reaction of Alexa Fluor™ 647 NHS-Ester (Thermo Fisher Science) or TAMRA-NHS with the antibodies was performed following the manufacturer’s protocol. Excess fluorescent dye was removed using Zeba™ Spin Desalting Columns, 7K MWCO (Thermo Fisher Science).

Antibody toxin conjugates were generated by an enzyme-mediated coupling reaction using a penta-mutated variant of sortase A [43] [eSrtA in pET29 (Addgene plasmid: # 75144)] [44]. For coupling of PE_cat_ and eGFP, the N-terminal fusion was cleaved off using TEV protease. Cleavage at an engineered ENLYFQG sequence between the two proteins to an accessible N-terminal glycine is required for the SrtA reaction.

After TEV protease cleavage was performed for 16 h at room temperature, for separation of cleaved (G_4_S)-eGFP and (G_4_S)_2_-PE_cat_ from the N-terminally MBP, respectively, subsequent Strep-Tactin ^®^XT purification was performed. The buffer was exchanged to SrtA buffer by dialysis overnight. Additionally, the C-terminals of the antibody heavy chains were genetically fused with the recognition sequence of SrtA (LPETGG). The coupling reaction was performed in 1× SrtA buffer (0.05 M Tris, 0.15 M NaCl, 0.005 M CaCl_2_, pH 7.5) conducted by 0.1 eq. SrtA Mut5 *E. coli* derived. The antibodies and (G_4_S)-eGFP and (G_4_S)_2_-PE_cat_, respectively, were applied to the reaction equimolar considering the number of coupling tags. For enhanced protein stability during the coupling reaction 2.5 mM (2-Hydroxypropyl)-β-cyclodextrin (Sigma–Aldrich) was added [45]. The coupling reaction was performed for 16 h at room temperature.

### 2.5. MMP-9 Cleavage

An MMP-9 recognition sequence (VHMPLGFLGP) was genetically inserted for TME-dependent cleavage of the masking unit from the cytosol-penetrating antibody. The cleavage was performed in sortase A or TN buffer (0.05 M Tris, 0.15 NaCl, pH 7.5) to provide optimal conditions for the MMP-9 cleavage reaction. Prior to cleavage, the recombinant human zinc-metalloproteinase (VWR, Avantor, Radnor, PA, USA) was pre-activated with 1 mM 4-aminophenylmercuric acetate (APMA) overnight at 37 °C. A total of 0.1 µg of MMP-9 was added to 0.1 mg of the respective masked antibody and incubated at 37 °C for 48 h. For PE_cat_-coupled antibodies, the coupling reaction was performed first, followed by cleavage with half of the reaction mixture. The mask and MMP-9 were not removed before the cell assays.

### 2.6. Confocal Microscopy

For visualization of the internalization of the cytosol-penetrating antibodies, they were coupled with (G_4_S)-eGFP, added to target cells in DMEM with 1% Penicillin/Streptomycin (Pen/Strep), and analyzed by confocal microscopy. Therefore, HeLa cells were seeded in an 18 well µ-slide (ibidi GmbH, Gräfelfing, Germany) with 8000 cells/well. One day post-seeding, the cells were treated with antibody-dye conjugate for 8 h and were washed three times with PBS afterwards. For visualization of the nucleus, the cells were incubated with 5 µg/mL Hoechst 33342 (H1399; Fisher Scientific, Hampton, NH, USA) in PBS for 10 min at room temperature, and then washed two times with PBS. For fixation, the cells were incubated in 4% paraformaldehyde for 20 min at room temperature. The samples were recorded in the 18 well slide with a Leica TCS SP8 confocal microscope (Leica Microsystems GmbH, Wetzlar, Germany).

### 2.7. Heparin Competition Assay

A heparin competition assay was performed for the determination of heparan sulfate proteoglycan binding-dependent internalizing capabilities of the cytosol-penetrating antibodies. Therefore, HeLa cells were seeded in a 96 well plate with 4 × 10^4^ cells/well. One day post-seeding, the cells were treated with 300 units/mL Heparin (H3149-10KU; Sigma–Aldrich) for 30 min, and were then washed three times with PBS before the fluorophore-coupled samples in DMEM with 1% Pen/Strep were added. After an 8 h incubation time, the cells were washed, trypsinized, and subsequently analyzed using the CytoFLEX S System (Beckman Coulter, Brea, CA, USA).

### 2.8. Internalization Assay

Receptor-mediated antibody internalization assays were performed using the pHAb Amine Reactive Dye (Promega, Madison, WI, USA) according to the manufacturer’s instructions. For this, SKBR-3 cells were seeded in a 96 well plate with 4 × 10^4^ cells/well. One day post-seeding, the cells were treated with antibody–pH dye conjugate in different concentrations (30–750 nM) in serum-free medium for 24 h at 37 °C and 5% CO_2_. The cells were then washed three times with PBS, trypsinized, and analyzed using the CytoFLEX S System (Beckman Coulter).

### 2.9. Cell Proliferation Assay

A PE_cat_-dependent cell proliferation assay was performed by a colorimetric method using CellTiter 96^®^ AQ_ueous_ One Solution Cell Proliferation Assay (Promega) for discrimination between endosomal and cytosolic localization of the antibodies in the cell. Therefore, the cells were seeded in 96 well plates with 4–8 × 10^3^ cells/well. One day after seeding, the cells were treated with the antibody–PE_cat_ conjugates in concentrations ranging from 0.3 nM to 500 nM in serial dilution in serum-free medium for 24 h. Afterwards, the medium was supplemented with 10% FBS and the cells were incubated for a further 48 h. After an overall incubation time of 72 h, the cells were supplemented with MTS solution according to the manufacturer’s instructions. The absorbance was measured at 490 nm using the CLARIOstar plus microplate reader (BMG LABTECH, Ortenberg, Germany). Mean values were determined from the cell proliferation data and normalized to the untreated controls. The resulting curves were plotted using GraphPad Prism over a variable slope four-parameter fit, and EC_50_ values were determined where possible.

### 2.10. Cell Binding Assay

For the determination of cell binding affinities of the analyzed antibodies to SKBR-3 cells, 2.5 × 10^5^ viable cells were seeded per well in a 96 well round-bottom plate and subsequently washed with 0.1% BSA-PBS by centrifugation at 800× *g*, at 4 °C and for 4 min. The cells were treated with the antibodies in a dilution series ranging from 15 nM to 4000 nM for 45 min at 4 °C. Afterwards, the cells were washed three times with 0.1% BSA-PBS and 25 µL of the secondary antibody, Goat anti-Human IgG Fc eBioscience™ PE (Invitrogen), was applied to the cells as 1:75 dilution. After a 20–30 min incubation time on ice, the cells were washed three times with 0.1% BSA-PBS und subsequently analyzed using CytoFLEX S System (Beckman Coulter).

To test for non-specific, HSPG-independent binding of native and masked CPAb on cells, HSPG-negative cells were prepared via incubation with 3 units/mL Heparinase I and III mix (H3917, Sigma–Aldrich). For this, 1.5 × 10^7^ HeLa cells were incubated in DMEM + 1% Pen/Strep supplemented with 15 units of heparinase for 30 min at 25 °C. Cell binding assays were then performed as described above.

### 2.11. NanoBiT Luciferase Assay

The NanoBiT luciferase assay (Promega) is a split luciferase-based assay, here consisting of an inducible LgBiT expressing HeLa cell line and HiBiT peptides, genetically fused to the C-terminus of the antibody heavy chain. Using a cell-penetrating furimazine-derived substrate Nano-Glo^®^ Vivazine™ (Promega) enabled the live cell monitoring of the cytosol-penetrating antibodies without prior cell lysis. Therefore, HeLa 11ht_LgBiT cells were seeded in a 96 well plate with 1.5 × 10^4^ cells/well. After 24 h, the cells were treated with HiBiT-fused antibodies or with the HiBiT peptide only in DMEM (Thermo Fisher Scientific) supplemented with 1% Pen/Strep (Thermo Fisher Scientific) in different concentrations (2–500 nM) and incubated at humidified conditions for 24 h. The medium was removed, and the cells were incubated with DMEM with 10% FBS, 1% Pen/Strep and, additionally, 1× NanoGlo^®^ Vivazine™ (Promega). After the two-hour incubation period, luminescence intensities of the samples were measured using the CLARIOstar plus microplate reader (BMG LABTECH). Mean values and standard deviation were determined from luminescence data and plotted using GraphPad Prism.

### 2.12. Synthesis of HiBiT Peptide

The HiBiT peptide (CGSSG-VSGWRLFKKIS-*NH*_2_) synthesis with additional N-terminal CGSSG, for further functionalization, if necessary, was performed on a 0.25 mmol scale using Amphispheres 40 RAM resin, and 0.46 mmol/g loading (Agilent Technologies, Waldbronn, Germany) via Fmoc-based solid phase peptide synthesis. The peptide was synthesized on an automated platform with microwave assistance using the LibertyBlue^®^ (CEM, Matthews, NC, USA). Fmoc-deprotection was performed with 20% piperidine (*v*/*v*) and 0.1 M ethyl 2-cyano-2-(hydroxyimino)acetate (oxyma) (Carbolution, St. Ingbert, Germany) in N,N-dimethylformamide (DMF) (Carl Roth, Karlsruhe, Germany). Amino acid activation and coupling was performed with N,N-diisopropylcarbodiimide (DIC) (Carbolution) and oxyma in DMF. The peptide was cleaved from the resin by treatment of the resin with trifluoracetic acid (TFA) (Carl Roth)/triethyl silane (TES) (Sigma–Aldrich)/Anisole (Thermo Fischer Scientific)/H_2_O (94/2/2/2) for 3 h and precipitated in cold diethyl ether (Honeywell, Charlotte, NC, USA). The peptide was dissolved in water, lyophilized, and purified by preparative reversed phase-high performance liquid chromatography (RP-HPLC). The product was analyzed via liquid-chromatography mass-spectrometry (LC-MS) and analytical RP-HPLC. A white product with a yield 167.2 mg (39%) was obtained.

### 2.13. Chicken Immunization

The CPAb was reformatted as scFv for in-house expression and purification from *Escherichia coli* (NEB: SHuffle T7 Express), according to Hinz et al., 2020 [46]. The following steps were performed by Davids Biotechnologie GmbH (Regensburg, Germany): In total, one chicken (*Gallus gallus domesticus*) was immunized five times with CPAb scFv on days 1, 14, 28, 42, and 56. After the 4th immunization, the titer was determined via ELISA. Spleen cell isolation and subsequent RNA extraction were performed after 63 days.

### 2.14. Yeast Library Generation and Sorting

Preparation and cloning of scFvs from chicken spleen cell RNA was performed as previously described [47]. In brief, cDNA was synthesized according to the SuperScript III Reverse Transcriptase (Invitrogen: 1808051) protocol. The product was used as a template to amplify V_H_ and V_L_ sequences by PCR with framework specific primers. Subsequent overlap PCR allowed fusion of the amplicons via a (G_4_S)_3_ linker. A modified pCT plasmid for yeast surface display for C-terminal protein display on Aga2p with tGFP expression as induction control [48] was prepared for homologous recombination (gap repair) by enzymatic cleavage with *NheI* and *BamHI* (R3131, R3136; NEB, Ipswich, MA, USA). Following the protocol of Benatuil et al. [41], scFv and backbone DNA were transformed by electroporation into *Saccharomyces cerevisiae* EBY100 [MATa URA3-52 trp1 leu2Δ1 his3Δ200 pep4::HIS3 prb1Δ1.6R can1 GAL (pIU211:URA3)] (Thermo Fisher Scientific). After regeneration, a serial dilution was plated on agar plates to calculate transformation efficacy, and thus to estimate the number of individual clones. The remaining cells were incubated in 1 L glucose containing SD medium for 24–48 h at 30 °C and 200 rpm. As described before [48], the resulting yeast library was inoculated overnight in 1 L galactose containing SG medium to induce expression of Aga2p-scFv. Level of surface presentation was indirectly detected by the intracellular fluorescence of tGFP produced after 2A-mediated ribosomal skipping on the same transcript. Before fluorescence activated cell sorting (FACS), cells were washed twice with phosphate-buffered saline containing 0.1% bovine serum albumin (PBSB, pH 7.4). As an antigen, CPAb was conjugated with Alexa Fluor 647 NHS Ester (Invitrogen) following the manufacturer’s protocol. CPAb-AF647 concentrations of 0.1–1 µM were prepared in PBSB and used for staining (20 µL per 1 × 10^7^ cells) for 30 min at room temperature, followed by washing and resuspension in PBSB. Sorting rounds were performed with the influx v7 sorter (BD, Franklin Lakes, NJ, USA, FACS software 1.0.0.650), with green and red fluorescence representing surface presentation and target binding, respectively. The outcome of each round was incubated on agar plates (48–72 h at 30 °C), followed by complete resuspension for subsequent induction and sorting, or by inoculation of individual colonies. Staining of single clones was performed as described above and cell cytometry was performed with the CytoFLEX S device (Beckman Coulter, CytExpert software 2.4.0.28).

### 2.15. Reformatting and Production of scFv-CPAb Fusions

Chicken-derived anti-CPAb scFvs were fused to the N-terminus of the light chain of CPAb via golden gate cloning into modified pTT5 mammalian expression vector containing *SapI* sites (R0569; NEB). Amplification and extension with the MMP-9 cleavable linker by Geiger et al. [35] were performed with the primers Chicken_VH_GG_up (5′-ATATATGCTCTTCAAGTGCTAGCGCCGTGACGTTGGAC-3′) and Chicken_VL_MMP-9_lo (5′-ATATATGCTCTTCAGAAGCCCAGGGGCATGTGCACGCTACCGCCGCCACCGCTGCCACCACCGCCTAGGACGGTCAGGGTTGTCCC-3′). Production in Expi293F™ HEK cells and purification via protein A affinity chromatography was performed as described before. Elution fractions were dialyzed against TN buffer (50 mM Tris, 150 mM NaCl, pH 7.4) and stored at 4 °C.

### 2.16. Size Exclusion Chromatography

Protein samples (40 µg) were analyzed in TN buffer (50 mM Tris, 150 mM NaCl, pH 7.4) on the TSKgel SuperSW3000 column (Tosoh Bioscience GmbH, Griesheim, Germany). Using the Agilent Technologies 1260 Infinity system, a constant flow rate of 0.35 mL/min was applied and protein elution was monitored at 280 nm.

## 3. Results

### 3.1. Identification of an Internalizing Antibody and Characterization of a CPAb-Binding Motif

**Figure 2:**
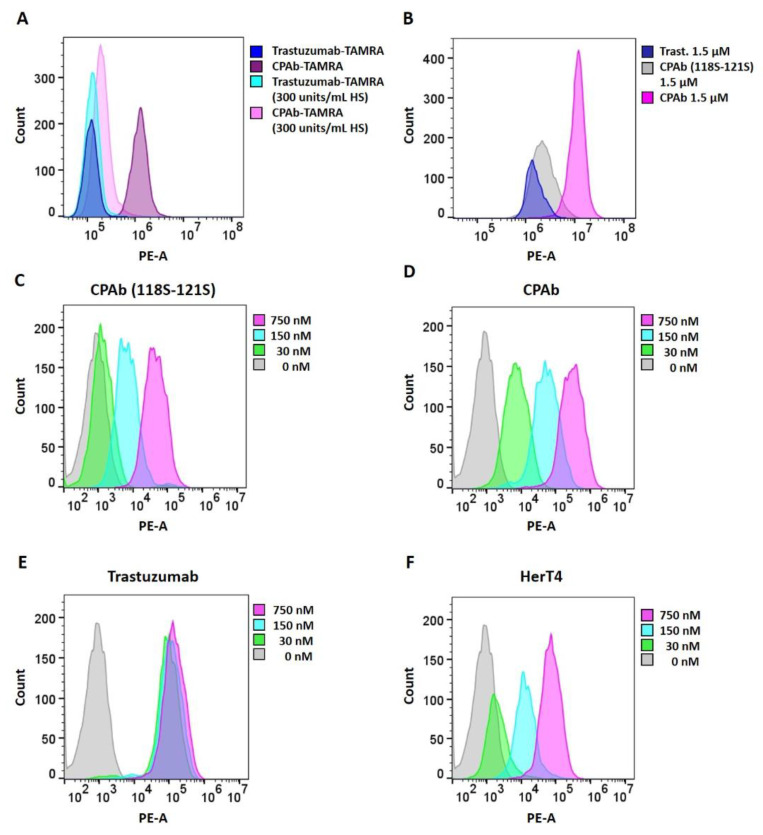
HeLa cell binding and internalization of CPAb, CPAb (118S–121S) and trastuzumab in HeLa and SKBR-3 cells. A total of 10,000 events per sample were recorded with the CytoFLEX S cytometer. TAMRA-labeled antibodies and internalized pH-dye labeled antibodies were detected in the PE channel. (**A**) In the heparin competition assay, HeLa cells were treated with 300 units/mL heparin sodium salt (HS) prior to antibody treatment. Either 2 µM TAMRA-coupled CPAb or trastuzumab, serving as a negative control, were added. (**B**) For investigation of the binding motif, TAMRA-coupled CPAb (118S–121S), CPAb (as positive control) or trastuzumab (as negative control) were added to HeLa cells. Fluorescence was measured 8 h after treatment. The internalization into SKBR-3 cells of (**C**) CPAb (118S–121S), (**D**) CPAb, (**E**) Trastuzumab and (**F**) HerT4 using a pH-dependent dye was analyzed after 24 h. The determined dye-to-antibody ratios ranged from 6.7–6.9. Untreated SKBR-3 cells were used as negative controls.

As part of a yeast display antibody library screening campaign to identify antibodies that target liver tumor cells, we discovered an antibody that displayed excellent liver cell binding. Unexpectedly, after reformatting from a Fab to a human IgG antibody, the purified TAMRA-labeled antibody, later named CPAb, has been observed to exhibit binding to liver tumor cells, as well as to a wide variety of tumor cell lines, including HeLa cells (Figure 2A). At least one order of magnitude higher binding was observed, compared to TAMRA-labeled trastuzumab, which served as a non-binding isotype control since trastuzumab binds to HER2, which is poorly expressed on HeLa cells [49,50]. Upon inspection of the CDR sequences of the heavy and the light chains of CPAb, the presence of the HSPG-binding motif ARRRRH (Ala117–His122) in the CDR3 of the heavy chain was indicated. To verify HSPG-dependent binding, a heparin competition assay was performed by measuring HeLa cell binding in the presence and absence of 300 units/mL heparin sodium salt (HS) and 2 µM antibody. Pre-incubation, and thus competitive binding to heparin, led to a significant decrease (10-fold) in fluorescence intensity, approaching the level of the trastuzumab isotype control. This corroborates the notion that CPAb contains a functional HSPG-binding motif that enables binding to a variety of cell lines. To confirm the suspected HSPG-binding motif being located in CDR-H3, a mutant variant of CPAb was generated, in which the basic amino acids Arg118 to Arg121 were substituted to serine (CPAb 118S–121S). Confirming the importance of the putative binding motif, the binding of the antibody to HeLa cells was impaired by these mutations, compared to the native CPAb variant (Figure 2B). Finally, binding assays were performed with HSPG positive and negative HeLa cells. The latter were generated via enzymatic degradation of heparin sulfate chains to test for non-specificity of CPAb towards a HSPG-negative cell surface (Figure S1). As expected, we observed a significant reduction in the MFI signal, by ~96%, for native CPAb on the negative cell line, compared to the native HSPG-positive cells.

For further characterization, we used SKBR-3 cells overexpressing HER2. Trastuzumab, a well-characterized HER2 binder, is known for its rapid and efficient endosomal uptake in SKBR-3, and was therefore used as a positive control in internalization assays [51]. To investigate internalization, CPAb wildtype, CPAb (118S–121S), and trastuzumab were fluorescently labeled with the pH-sensitive pHAb amine reactive dye. This dye displays minimal fluorescence at neutral pH but enhanced fluorescence at acidic pH, and is therefore routinely used as an endocytosis tracker [51].

SKBR-3 cells were incubated with antibody–pH dye conjugates at different concentrations (30 nM, 150 nM, 750 nM) for 24 h. Concentration-dependent cell internalization could be confirmed for the native CPAb antibody (Figure 2D) and for trastuzumab (Figure 2E). However, an increase in internalization was not observed within the tested concentration range for trastuzumab, most likely due to receptor saturation at 30 nM (Figure 2E). The CPAb (118S–121S) variant lacking the HSPG-binding motif showed significantly reduced internalization, compared to wildtype CPAb at all three concentrations tested (Figure 2C,D). This is consistent with in vitro cell binding data using TAMRA-labeled antibody (Figure 2B). Finally, for comparison, the cytosol-internalizing antibody HerT4 cytotransmab was pH dye-labeled. As expected, internalization of HerT4 cytotransmab was also observed (Figure 2F). However, the mean fluorescence intensity was significantly reduced, compared to CPAb in the tested concentration range, even though both antibodies displayed similar binding curves in an in vitro binding assay on SKBR-3 cells (Figure S2). These results indicate that HerT4 cytotransmab has a lower internalization efficiency, compared to CPAb.

### 3.2. Confocal Microscope Imaging

**Figure 3:**
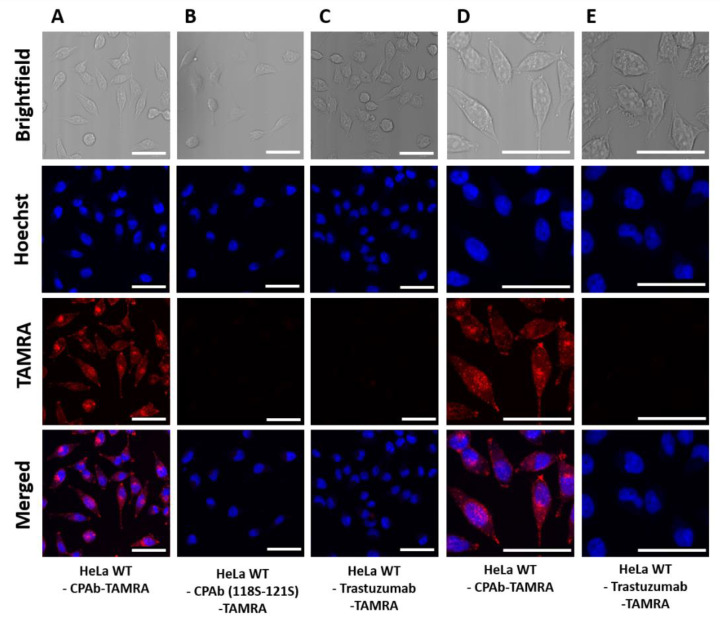
CLSM images as brightfield images or TAMRA fluorescence images of HeLa wildtype cells with (**A**) 1.5 µM CPAb-TAMRA, (**B**) 1.5 µM CPAb (118S–121S)-TAMRA, or (**C**) 1.5 µM trastuzumab-TAMRA. (**D**,**E**) show 2-fold magnifications of cells treated with CPAb-TAMRA or trastuzumab-TAMRA, respectively. The white scale bar is equivalent to 50 µm. Fluorescence images were generated using ImageJ 1.53c.

The cellular distribution of the respective antibodies after internalization was analyzed using confocal laser scanning microscopy (CLSM) (Figure 3). HeLa cells were treated with 1.5 µM TAMRA-NHS coupled antibodies, including CPAb wildtype, CPAb (118S–121S), and trastuzumab for 8 h at 37 °C. As expected, HeLa cells treated with CPAb (118S–121S) and trastuzumab, serving as isotype control, did not exhibit any TAMRA fluorescence in the cells. However, CPAb-TAMRA resulted in a distinctive homogeneous distribution of TAMRA fluorescence in the HeLa cells, in contrast to the trastuzumab control (Figure 3D,E). The mean fluorescence was additionally determined using flow cytometry for qualitative evaluation (Figure S3). Comparison with HerT4 cytotransmab was not feasible, due to repetitive protein aggregation after TAMRA-NHS labeling.

**Figure 4:**
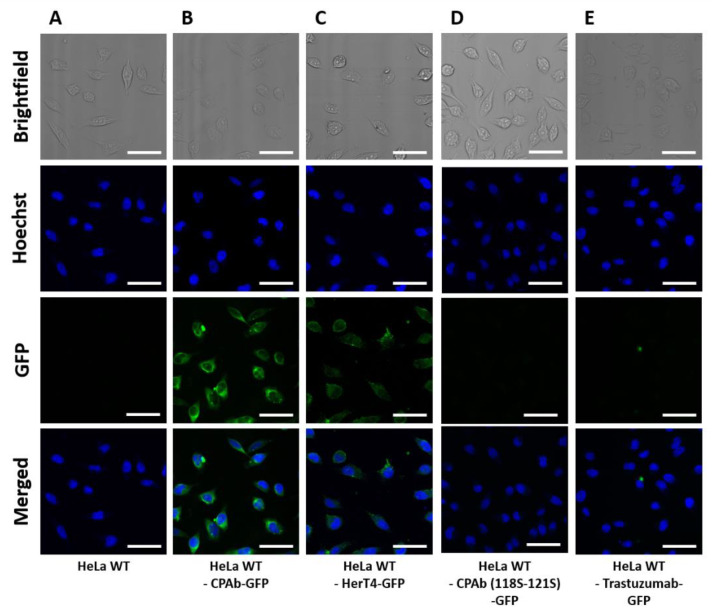
CLSM images as brightfield images or GFP fluorescence images of HeLa wildtype cells (**A**) untreated or treated with (**B**) 1 µM CPAb-GFP, (**C**) 1 µM HerT4-GFP, (**D**) 1 µM CPAb-(118S–121S)-GFP, and (**E**) Trastuzumab-GFP. The white scale bar is equivalent to 50 µm. Fluorescence images were generated using ImageJ 1.53c.

To investigate if the internalizing antibody is able to deliver a protein cargo to the cell cytoplasm, antibody-eGFP conjugates were generated through a sortase A-mediated coupling reaction. Therefore, CPAb and trastuzumab were endowed with a LPETGG sortase A recognition sequence at the C-terminus of the heavy chain. Additionally, enhanced green fluorescent protein (eGFP) [52] containing an N-terminal triple glycine sequence was used for sortase A (eSrtA) ligation [43,53]. The reaction efficiency was analyzed using SDS-PAGE (Figure S4A), indicating a coupling efficiency of approximately 70%. HeLa cells were treated with 1 µM antibody-GFP conjugate for 8 h. For the quantitative investigation, the mean fluorescence intensities were determined via flow cytometry (Figure S4B). As expected, HeLa cells treated with trastuzumab-eGFP did not exhibit GFP fluorescence (Figure 4E). A diffuse fluorescence was observed in cells treated with CPAb-eGFP, and also with HerT4 cytotransmab-eGFP, being distributed throughout the cell, except for the nucleus (Figure 4B,C). Upon closer examination, however, a punctuate fluorescence pattern was detected for the HerT4-GFP conjugate, indicating partial localization in intracellular vesicles (e.g., early endosomes). This suggests partial endosomal membrane-penetration, and also incomplete release into the cytosol. Both qualitative CLSM and quantitative flow cytometry results indicate that CPAb-eGFP was internalized to a greater extent than the HerT4 cytotransmab-eGFP conjugate (see Figure S4B). Additionally, CPAb (118S–121S)-eGFP did not show any cytoplasmic localization (Figure 4D).

### 3.3. Investigation of In Vitro Cytosol-Penetration Capabilities

#### 3.3.1. PE_cat_-Mediated Cell Proliferation Assay

**Figure 5:**
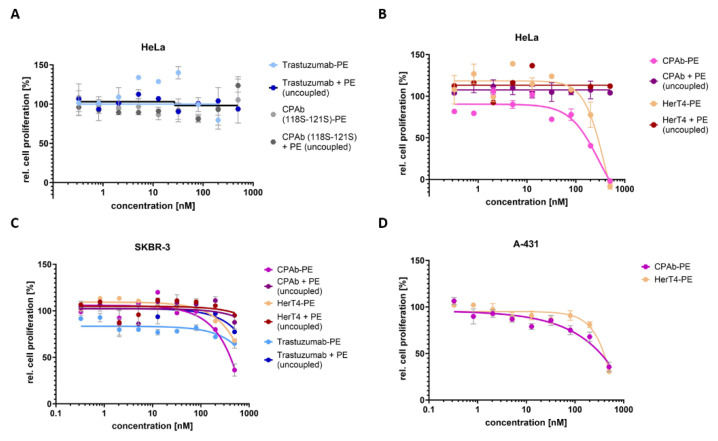
Proliferation assays of CPAb and HerT4, in comparison to several negative controls on HeLa, SKBR-3 and A-431 cells. The antibodies, coupled or uncoupled, were added to the cells for 72 h in a dilution series ranging from 0.2 to 500 nM. The resulting data points are shown as mean and error bars that represent standard deviation derived from experimental duplicates or triplicates. EC_50_ values were determined from variable slope four-parameter fitting using GraphPad Prism 10.1.0 (316). (**A**) The negative controls trastuzumab and mutated variant CPAb (118S–121S) were tested, coupled, and uncoupled on HeLa cells. (**B**) The cytosol-penetrating antibodies CPAb and HerT4 were analyzed as PE_cat_ conjugate, resulting in EC_50_ values of 200 nM or 300 nM, respectively. (**C**) Cytosol-penetration of coupled and uncoupled CPAb-PE_cat_ and HerT4-PE_cat_ were further tested in comparison with internalizing trastuzumab-PE_cat_, using SKBR-3 cells, and not resulting in a determinable EC_50_ value. (**D**) Proliferation assay of CPAb-PE_cat_ and HerT4-PE_cat_ in A-431 cells.

To investigate if CPAb is released from the endosome to the cytosol after receptor-mediated internalization, together with an attached cargo protein, we replaced eGFP with a truncated version of a 24 kDa fragment of *Pseudomonas* exotoxin (PE24) [37]. The catalytic ADP ribosylation domain of PE24 contains a C-terminal recognition sequence (KDEL) for retrograde transport to the cytoplasm [37,54]. Once in the cytoplasm, the catalytic domain modifies the elongation factor 2 (EF-2), resulting in the inhibition of translation and ultimately leading to cell death [54]. It is important to note that PE24 lacking the C-terminal signal sequence (KDEL) is unable to reach the cell cytoplasm [38,55,56]. Thus, we removed the translocation sequence from PE24, resulting in the truncated ‘PE_cat_’ version. If the truncated PE_cat_ is able to reach the cytosol upon antibody conjugation, reduced cell viability would indicate successful cytoplasmic cargo delivery. To assess this, we designed a proliferation assay dependent on PE_cat_, which involves conjugating a cell-penetrating antibody to the toxin’s catalytic domain (Figure 1). This allowed detection of internalization without the need for stably transfected reporter cells, and thus conjugates can be tested universally on several cell lines. Therefore, PE_cat_ was coupled to CPAb and HerT4 cytotransmab via a sortase A-mediated reaction. On average, about 60% of the conjugate was obtained, resulting in a heterogeneous mixture of antibodies, coupled with 0, and up to 2 toxin units (Figure S5A,B). CPAb (118S–121S) was used as a non-internalizing control, while trastuzumab served as the reference molecule, being internalizing but not cytosol-penetrating [57,58]. To exclude false positive results caused by proliferation-inhibiting effects mediated by the individual proteins, we tested the PE_cat_ toxin alone, and the respective uncoupled antibody were tested as additional controls. Neither CPAb (118S–121S) nor trastuzumab, whether conjugated with PE_cat_ or not, showed a cell proliferation inhibitory effect on HeLa cells (Figure 5A). Trastuzumab-PE_cat_ exhibited a proliferation inhibitory effect on HER2 positive SKBR-3 cells at concentrations of 200 nM and above, resulting in a decrease of 27% in viable cells (from 92% to 65%). This effect can be attributed to the inhibitory effect of unconjugated trastuzumab on SKBR-3 cells at higher concentrations, as generally observed (Figure 5C). Subsequently, CPAb, as well as the HerT4 cytotransmab, were tested on HeLa, SKBR-3, and A-431 cells in dilution series starting at a concentration of 500 nM (Figure 5B–D). CPAb, as well as HerT4 cytotransmab, and PE_cat_, as uncoupled controls, did not significantly inhibit cell proliferation in HeLa and SKBR-3 cell lines at the highest concentration tested (Figure 5B,C). The CPAb-PE_cat_ conjugate, however, exhibited a concentration-dependent cytotoxic effect in all three cell lines, with an EC_50_ value of 200 nM on HeLa cells and a maximum decrease of viable cells of 65% on A-431 and SKBR-3 cells at 500 nM. The HerT4 cytotransmab-PE_cat_ conjugate showed a cell-killing effect in HeLa and A-431 cells only at the highest concentration of the dilution series. The EC_50_ value on HeLa cells was determined to 300 nM, and the maximal killing efficiency to 70%. However, the cytotoxicity study using SKBR-3 cells resulted in only a 32% decrease in viable cells. In summary, the results of the in vitro cytotoxicity studies indicate that the CPAb described here has an improved ability for cytosol penetration into different cell lines and for delivering cargo proteins into the cytosol, compared to the previously published HerT4 cytotransmab antibody.

#### 3.3.2. Split Luciferase-Based Cytosol-Penetration Assay

**Figure 6:**
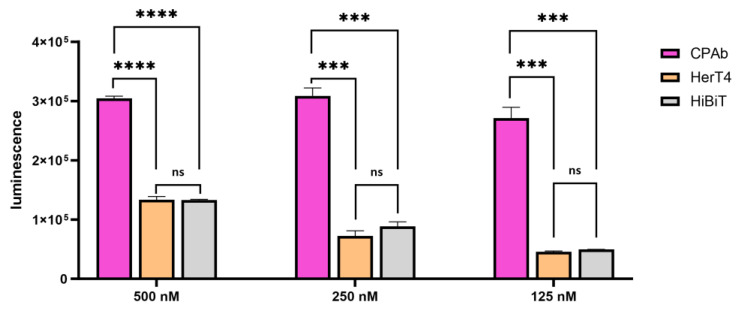
NanoBiT assay for cytosol-penetrating studies of CPAb-HiBiT_2_ and HerT4-HiBiT_2_, in comparison to HiBiT peptide in different concentrations (500 nM, 250 nM, and 125 nM). The concentration of the HiBiT peptide was adjusted to the amount of peptide per antibody, resulting in 2-fold higher concentrations. The results were shown as mean values, with error bars representing the standard deviation resulting from experimental duplicates. One-way ANOVA with Tukey’s multiple comparisons tests (with *p* value style GP: 0.1234 (ns), 0.0002 (***), and <0.0001 (****)) were used to display the significance level (with definition of statistical significance: *p* < 0.05). Statistical analysis was performed in GraphPad Prism 10.1.0 (316).

For validation of the cytosol penetration studies, we investigated the cytosol location of CPAb using the NanoBiT Assay (Promega). The NanoBiT Assay is a split luciferase-based assay including a 17.9 kDa inactive enzyme (LgBiT) and the HiBiT peptide consisting of 11 amino acids [59]. Cell supplementation with a furimazine-derived substrate allows for the detection of intracellular complementation of the HiBiT peptide and the intracellularly-produced inactive LgBiT through luminescence measurement [60]. To achieve this, we generated a stable HeLa cell line with inducible LgBiT expression. The NanoBiT assay was performed using antibodies with genetically integrated HiBiT at the C-terminus of the heavy chain. Vivazine was utilized as a cell permeable substrate in these studies. In the NanoBiT cytosol penetration assay, CPAb-HiBiT_2_, HerT4 cytotransmab-HiBiT_2_, and the HiBiT peptide (peptide analysis: Figures S6 and S7) were tested at varying concentrations (Figure 6). In this cytoplasmic complementation assay, CPAb-HiBiT_2_ demonstrated a significant increase in luminescence intensity, compared to the solitary peptide at all tested concentrations. In contrast, when examining the HerT4 cytotransmab-HiBiT_2_ samples, significantly lower luminescence intensities were observed, in comparison to CPAb-HiBiT_2_. These intensities were in the range of the solitary peptide alone, which supports the notion that CPAb displays better complementation of the truncated split luciferase, and thus better cytosolic penetration after 24 h.

### 3.4. Generation and Characterization of Conditionally Masked CPAb

To generate an affinity-based masking domain directed against the paratope of CPAb, chicken immunization was performed with an in-house produced antigen (Figure S8). For this, the CPAb was reformatted and used as scFv for immunization to focus the immune response to the variable domains, and a high antibody titer was obtained after the fourth booster immunization (Figure S9). After the fifth booster, the chicken was sacrificed on day 63 and the RNA was isolated from spleen cells. cDNA synthesis was performed via RT-PCR, and chicken scFvs were generated by two PCR steps. An immune library was generated from the chicken-derived scFvs by transformation with a yeast surface display plasmid in *Saccharomyces cerevisiae*, resulting in a library diversity of 1.3 × 10^9^ transformants. The scFv library underwent four rounds of screening (Figure S10A–E) with Alexa Fluor 647 labeled CPAb (Figure S10F) via fluorescence activated cell sorting (FACS). A high antigen concentration of 1 µM CPAb-AF647 was selected to obtain antibodies with a broad spectrum of affinities, as we have found in previous studies that high affinity masks can prevent full recovery of antibody function, resulting in insufficient fold activation [61]. As demonstrated in Figure S10C, staining with 100 nM target revealed the presence of a subset of clones with higher affinity in the second round outcome. After the fourth round, ten single clones were tested with 1 µM CPAb-AF647, and clone S5 exhibited the highest apparent affinity (Figure S11A). In total, six clones were sequenced and, surprisingly, clones S4 and S6 consisted solely of the V_L_ and linker sequences (Figure S12). To investigate masking capabilities, we selected the full-length scFv clone 5 (241 AA, 24.5 kDa, pI: 4.6) and the significantly smaller V_L_-only antibody fragment, clone 4 (138 AA, 13.9 kDa, pI: 4.1), for reformatting as an N-terminal light chain fusion onto CPAb. We implemented a protease-cleavable linker, as described by Geiger et al. [35], for conditional activation by cell-secreted proteases or by manual addition of MMP-9 prior to functional assays. In the initial experiment to assess masking capability, 100 nM of scFv-CPAb fusions were tested for binding on HeLa cells for 30 min on ice (Figure S11B). Compared to a non-binding scFv genetically fused to CPAb as an isotype control, the fusion of S4 or S5 to CPAb resulted in a significant reduction in cell binding (Figure S11B). Non-specificity of unmasked antibodies was evaluated for S4-CPAb in a binding assay with HSPG- positive and -negative cells. HSPG degradation resulted in a ~95% MFI reduction for MMP-9-cleaved S4-CPAb at the highest concentration of 2 µM, thus abolishing binding.

**Figure 7:**
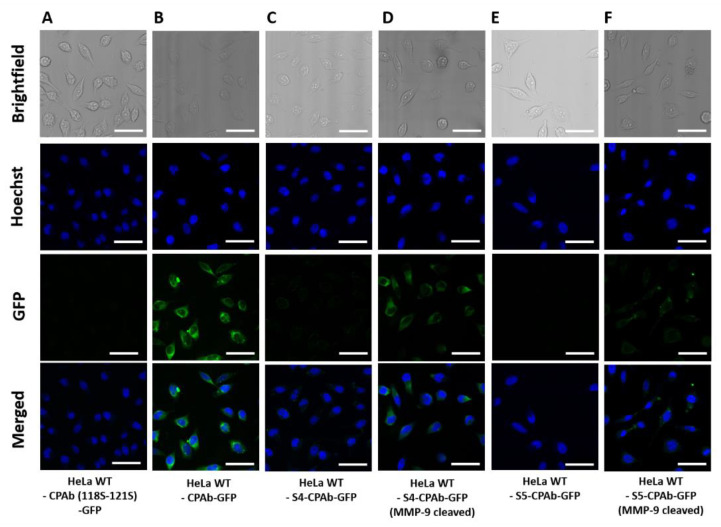
CLSM images of the brightfield or GFP fluorescence channels of HeLa cells treated with 1 µM antibody–GFP conjugates. (**A**) CPAb (118S–121S)-GFP, (**B**) CPAb-GFP, (**C**) S4-CPAb-GFP, (**D**) S4-CPAb-GFP (MMP-9 cleaved), (**E**) S5-CPAb-GFP and (**F**) S5-CPAb-GFP (MMP-9 cleaved) were incubated for 8 h with HeLa cells and subsequent washing and fixation with 4% PFA. For the GFP fluorescence imaging, the laser with 488 nm was utilized. The scale bar is equivalent to 50 µm. Fluorescence images were generated using ImageJ 1.53c.

For investigating the capability of cytoplasmic internalization, the potentially masked antibodies were conjugated with eGFP using sortase A-mediated reaction (Figure S3A). Cellular localization was investigated by confocal microscopy after 8 h antibody incubation at 37 °C with Hela cells. For comparison, unmasked CPAb and CPAb (118S–121S) are shown in Figure 7A,B. No intracellular fluorescence was detected for the masked variants S4-CPAb and S5-CPAb, indicating that the mask acts as an inactivating agent for antibody binding and cytosolic penetration (Figure 7C,E). In contrast, MMP-9 cleavage of S4-CPAb-eGFP (Figure 7D), showed a similar appearance in the CLSM images, as CPAb-eGFP without prior masking (Figure 7B). However, S5-CPAb after MMP-9 cleavage showed a punctate fluorescence pattern in the cells, compared to both S4-CPAb (unmasked) and native CPAb, indicating incomplete activation of the antibody after MMP-9 cleavage (Figure 7F).

**Figure 8:**
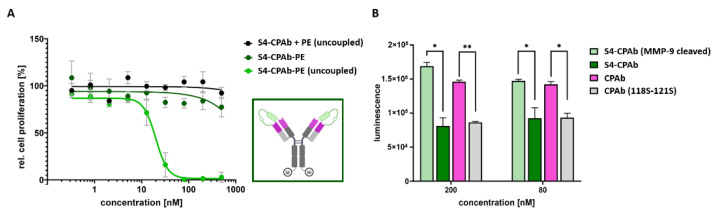
Cytosol-penetration capabilities of the masked S4-CPAb (MMP-9 cleaved and uncleaved). (**A**) Proliferation assay of S4-CPAb-PE_cat_ (MMP-9 cleaved und untreated), in comparison to unconjugated PE_cat_ and S4-CPAb in HeLa cells at different concentrations (0.2–500 nM). The resulting data points, shown as mean and error bars, represent standard deviation derived from experimental duplicates. EC_50_ values were determined from variable slope four-parameter fitting using GraphPad Prism 10.1.0 (316). (**B**) NanoBiT^®^ assay of S4-CPAb-HiBiT_2_ (MMP-9 cleaved und untreated), in comparison to CPAb-HiBiT_2_ and CPAb (118S–121S)-HiBiT_2_. The resulting data points, shown as mean and error bars, represent standard deviation derived from experimental duplicates. An unpaired, two-tailed *t*-test (with *p* value style GP: 0.1234 (ns), 0.0332 (*), 0.0021 (**)) was used to display the significance level (with definition of statistical significance: *p* < 0.05).

To further investigate the cytosol-penetrating capabilities of the masked CPAb variants, a cell proliferation study was performed. The masked antibodies were conjugated to PE_cat_ by sortase A, and subsequently half of the reaction mixture was cleaved with MMP-9 and no further purification for mask or MMP-9 removal was performed. Both reactions were analyzed by SDS-PAGE (Figure S15) and a coupling efficiency of ~60% was determined. The mask was completely cleaved by MMP-9 incubation (Figure S15). The different variations of masked CPAb variants (S4/S5-CPAb + PE (unconjugated), S4/S5-CPAb-PE, and MMP-9 cleaved S4/S5-CPAb-PE variants) were applied to HeLa cells in a concentration series ranging from 0.3 nM to 500 nM (Figure 8A and Figure S17). No significant inhibition of cell proliferation was observed in HeLa cells treated with unconjugated S4/S5-CPAbs up to 500 nM. Similarly, the masked S4-CPAb-PE did not exhibit any cytotoxic effects on the HeLa cells, indicating that the masking of the antibody fragment inhibited binding and cytosol penetration. In contrast, cleavage of the mask with MMP-9 resulted in cell killing with an EC_50_ value of 20 nM (Figure 8A). The experimental results for the S5 variants are comparable to those of the S4 variants. However, the unmasked S5 scFv induced cytotoxicity in Hela cells at higher concentrations, resulting in an EC_50_ value of 156 nM (Figure S17). Furthermore, SEC data revealed aggregation for S5-CPAb, which was not observed for S4-CPAb (Figure S14). Hence, our further studies focused on S4 masked CPAb.

Using the NanoBiT assay, the masked variants were analyzed by comparison with the native CPAb and the mutant CPAb (118S–121S) at different concentrations ranging from 5 nM to 500 nM (Figure 8B and Figure S16). The S4 masked CPAb-HiBiT_2_ exhibited luminescence at the level of the non-internalizing CPAb (118S–121S) variant, but MMP-9 cleavage resulted in a regain of luminescence close to the level of the native CPAb-HiBiT_2_. Thus, two orthogonal assays demonstrated the masking capabilities of S4 and the regeneration of cytosol-penetrating capabilities after cleavage.

**Figure 9:**
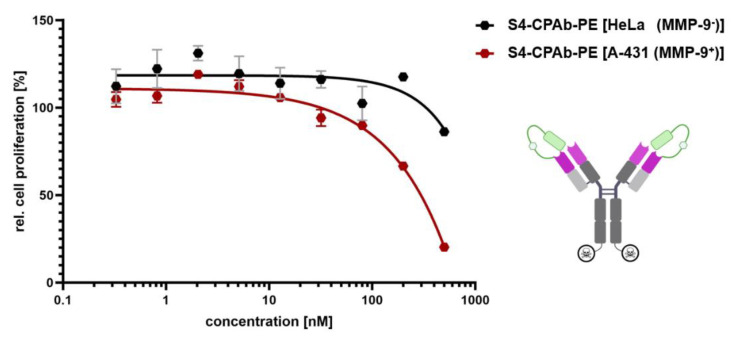
Proliferation assay for determination of cytosol-penetration capabilities of the masked S4-CPAb-PE in HeLa (MMP-9 negative) and A-431 (MMP-9 positive) cells at different concentrations (0.2–500 nM). The resulting data points, shown as mean and error bars, represent standard deviation derived from experimental duplicates using GraphPad Prism 10.1.0 (316).

Finally, a PE-mediated cell killing assay was performed to investigate if the conditional activation of S4-CPAb-PE_cat_ occurs through MMP-9 that is secreted by tumor cells. For this purpose, S4-CPAb-PE_cat_ was applied to HeLa cells (very low expression levels of MMP-9; [62]) and to A-431 cells (MMP-9 positive; [63]), respectively, in a dilution series (Figure 9). In MMP-9 secreting A-431 cells treated with S4-CPAb-PE_cat_, a significant cell-killing effect was observed, in contrast to HeLa cells. However, the maximum killing effect was only 80%, potentially due to incomplete cleavage of the mask. Consequently, MMP-9-dependent activation of CPAb by removal of the masking entity could be confirmed.

## 4. Discussion

Although protein binding entities for almost all cell surface or extracellular targets exist or can be easily isolated, functional tuning of intracellular targets by biologics is still limited, due to restricted access to the cell cytoplasm. Several cell-penetrating peptides (CPPs) have been described that gain access to the cytoplasm directly or via endosomal uptake routes [64]. However, their capability for delivery of peptide or protein cargoes, or for the use as protein transduction agents by co-administration of cargo proteins, is limited [64,65]. Specifically, their lack of cell type selectivity and low in vivo stability hampers their application [66,67]. Interestingly, pathogenic bacteria, such as *Pseudomonas aeruginosa*, have established routes for receptor-mediated endosomal uptake and retrograde transport into the cell cytoplasm [54]. The potential use of this pathway for cytoplasmic delivery while simultaneously addressing intracellular targets has only been described for a small set of cargo proteins [68,69,70]. Attempts have been made to induce cell-specific cytosol penetration through an endosomal uptake route, combined with the use of endosomal escape peptides (EEPs). These peptides enable pH-dependent cytosol penetration through the endosomal membrane by generating a “proton sponge effect” [71], which eventually leads to endosomal membrane leakage and cargo release into the cytoplasm [71,72,73]. Tumor cell targeting using these peptides was achieved by conjugation to tumor-specific antibodies, where receptor-mediated endocytosis of the antibody conjugate, followed by cleavage of the antibody, resulted in their endosomal escape to the cytoplasm [72].

Interestingly, several camelid nanobodies have been found that are able to reach the cell cytoplasm and specifically modulate intracellular protein functions [74,75]. Additionally, cell-penetrating antibodies have been discovered that bind to cell surface-exposed heparan sulfate proteoglycan, undergo endocytosis and endosomal escape, and eventually locate, at least in part, to the cytoplasm [22]. The best characterized internalizing antibody is TMab4, which displayed cell penetration even upon systemic administration in mice [22,25,76].

We serendipitously identified a similar cell-penetrating antibody, CPAb, while screening a scFv yeast display library. Upon reformatting as a full-length IgG, it was found to be located in the cytoplasm of HeLa cells. The presence of a tetra-arginine motif in the V_H_ CDR3 loop of CPAb is essential for cell binding and uptake, which was completely abolished by serine substitution at arginine 118–121 (Figure 2B). The addition of soluble heparin as a competitor (Figure 2A) or enzymatic removal of HS from the cell surface (Figures S1 and S13) also significantly reduced binding, supporting the notion that CPAb internalization is triggered by binding to cell surface HSPG, followed by endocytosis.

A mechanism has been proposed for TMab4 to allow for the escape of an internalized antibody into the cytosol. This antibody contains a HSPG-binding motif in the CDR1 of the light chain and a hydrophobic aromatic WYW motif in V_L_-CDR3 at position 92–94 [22,23]. It has been suggested that the residue AspL1 in the V_L_ framework region and MetL95 in V_L_-CDR3 act as a pH sensing pair. Acidification of the endosome is thought to result in protonation of Asp1, which eventually triggers local structural arrangements of MetL95 and thus of the WYW loop. This change promotes interaction with the endosomal membrane, ultimately leading to membrane lipid flip-flop and endosomal escape to the cytosol [23]. The endosomal escape motif could also be transferred to the V_H_ domain (95GWYWMDL102) of cytotransmab while retaining its internalization capability [24]. The CDRs of CPAb (sequence in Table S1) contain only one stretch of hydrophobic aromatic residues, which is located directly adjacent to the HSPG-binding tetra-arginine motif RRRRHFDYW. We speculate that this HFDYW motif, which also contains a His and Asp residue, can be protonated at acidic pH, thus inducing conformational changes that may mediate perturbation of the endosomal membrane, playing a major role in endosomal escape. Detailed mutation analysis of CPAb will be required to elucidate its endosomal escape mechanism.

In this study, we compared the cell-penetrating properties of CPAb with HerT4 cytotransmab, a chimeric antibody in which the endosomal escape mediating V_L_ domain (hT4) is paired with the heavy chain of trastuzumab [22]. The results demonstrate that both directly fluorescently labeled CPAb and a version with conjugated eGFP as a cargo were located intracellularly upon CLSM inspection (Figure 4). However, detecting the cytoplasmic location solely through direct or indirect fluorescence labeling or eGFP fusion of an antibody is insufficient, since it cannot be excluded that the antibody resides in the cytoplasm, in the form of membrane-enclosed vesicles, or that partial proteolytic degradation occurs.

Hence, for the discrimination of cytosolic or endosomal localization within the cell, two additional orthogonal assays were utilized: the PE_cat_ assay and the NanoBiT assay. To prove cytoplasmic localization, we generated antibody conjugates with the truncated catalytic domain of *Pseudomonas* exotoxin 24 (PE_cat;_ sequence in Table S1), without its own C-terminal motif that mediates retrograde transport into the cytoplasm [37,55,56]. When present in the cytoplasm, PE_cat_ catalyzes ADP ribosylation and induces cell killing. It is noteworthy that a mixture of CPAb and PE_cat_ had no effect on cell viability, whereas conjugation of PE_cat_ to the C-terminus of CPAb resulted in concentration-dependent cytotoxicity, supporting the notion that CPAb acts as a translocation vehicle for the PE_cat_ cargo (Figure 5). The EC_50_ values for the HerT4 cytotransmab (300 nM in HeLa cells) and the CPAb (200 nM in HeLa cells) were determined using the PE_cat_ derived proliferation assay. For comparison, tumor cell-targeting Fv-PE conjugates, such as the FDA and EMA approved moxetumomab pasudotox (Lumoxiti^®^), a CD22-binding immunotoxin containing a 38 kDa fragment of PE (PE38) with the C-terminal translocation sequence KDEL, achieved EC_50_ values in the picomolar range, significantly lower than the values mentioned in our study [77,78]. Differences in the efficiency of cellular uptake and endosomal escape, as well as the fact that we observed an incomplete conversion of CPAb into CPAb-PE_cat_ upon sortase A-mediated ligation, are most likely responsible for this difference.

An additional assay for determining cytoplasmic location involves delivering the HiBiT peptide to the cytosol to functionally complement truncated luciferase. It is important to note that caution should be exercised when interpreting results, as the luminescence signal may also be contributed by lysed cells that release luciferase. Additionally, luciferase complementation using a HiBiT peptide coupled to an antibody may be less efficient. However, we observed higher luminescence in this assay, compared to CPAb with the 118S–121S substitution. In all cytosol penetration assays conducted in this study, CPAb achieved results that were either equally good or better than the well-characterized HerT4 cytotransmab previously published, providing evidence of cytoplasmic localization.

Furthermore, we generated conditionally masked antibodies based on the CPAb. Therefore, we immunized a chicken with the CPAb scFv and isolated and tested CPAb binders after four rounds of fluorescence-activated cell sorting. Two suitable candidates were identified, which were fused to the CPAb light chain via a GS linker containing an MMP-9 cleavage site [35]. In particular, the masked version with the S4 variant showed similar results to the non-binding CPAb (118S–121S) in both assays, indicating excellent masking and prevention of cell binding. Notably both this V_L_ only antibody fragment S4 and the scFv S5 carry a large number of negatively charged Asp/Glu residues and display low pI values < 5.0 (Figure S12). Additionally, S4 also has a lower theoretical pI, compared to S5 (ProtParam: 4.1 vs. 4.6, respectively). Thus, despite the missing V_H_ domain, a presumed strong interaction of negatively charged residues in S4 with the positive charges in the CPAb paratope, indispensable for internalization capability, could explain the observed strong masking mediated by S4. When the S4-CPAb was cleaved with MMP-9, it resulted in an even more improved EC_50_ of 20 nM in the PE_cat_ cell-killing assay and similar luminescence intensities in the NanoBiT assay, compared to the native CPAb. Finally, it was demonstrated that the cleavage of the masking unit can occur exclusively through the secreted MMP-9 of tumor cells, using A-431 as an MMP-9 secreting cell line.

For cell-specific targeting of antibodies that locate to the cell cytoplasm, two routes can be considered. The first route involves replacing HSPG-binding functionality with targeting a tumor-specific cell surface receptor. Kim et al. [24] achieved this by engineering the TMAb4 cell-penetrating antibody. The HSPG-binding motif was removed, and an EpCAM-binding peptide sequence was fused to the heavy chain while retaining the endosomal escape motif. This strategy requires the careful design of receptor release in the acidic environment of the endosome by establishing relatively low affinity binding to the receptor and/or strongly reduced pH-dependent affinity for the receptor binding entity. The latter can be implemented by introducing histidine residues that become protonated upon acidification, resulting in repulsive forces upon receptor binding [79,80,81]. Here, we applied a different strategy that is based on fusing a masking scFv or V_L_ fragment to CPAb, which is linked to the antibody by a protease cleavable linker. Masking should result in shielding the internalizing antibody from HSPG interaction. Through library screening of an immune library from a CPAb-immunized chicken, we isolated a clone that lacked the connecting linker and heavy chain moiety of the intended scFv. However, it displayed excellent shielding of internalization as the V_L_ domain alone.

Regarding possible in vivo studies, we hypothesize that the native CPAb variant, without charge shielding of the paratope, would bind all cell types indiscriminately, due to the ubiquitous presence of HSPG on cell surfaces [12]; thus, we assume only a fraction would internalize and accumulate in desired target cells. Furthermore, we suspect that the CDR residues involved in electrostatic interactions with HSPG are prone to saturation and blocking via negatively charged serum components, resulting in low biodistribution and bioavailability of active antibodies in the organism. Consequently, we postulate that conditional masking of CPAbs is beneficial, or even inevitable, to minimize inactivation and potential side effects caused by off-tumor binding and internalization. Extrapolating from the comparatively high EC_50_ values of the antibodies determined in cell culture experiments, it might be necessary to administer unusually high doses during in vivo studies in order to demonstrate functionality of CPAb–effector fusions. These high concentrations might have the potential to increase possible toxic and immunogenic effects. Hence, we argue that implementation of antigen-specific cell binding capabilities is beneficial or even may be essential for in vivo experiments. Conceptually, this can be achieved by combining cell-specific targeting with CPAb-mediated cytosolic uptake of cargoes to potentially increase internalization rate while minimizing potential side effects of cell-type unspecific binding.

To the best of our knowledge, this is the first example of an antibody that reaches the cytoplasm and can be conditionally activated in the tumor microenvironment to regain its cytosol-penetrating capabilities. This strategy for antibody activation is cell-type agnostic, with the advantage that such a masked CPAb can be used to target different tumors without further modification, thus allowing different tumor types to be targeted by the same antibody. Obvious potential applications are TME-dependent cytosolic delivery of nanobodies, single chain variable fragments, affibodies, DARPins, and other protein-based entities that bind to intracellular pathologically altered proteins (e.g., against BCR-ABL oncogenic protein [82], RAS [83], or LMO2 [84]) in cancer cells. It remains to be examined whether, in addition to tumor-specific translocation of protein-based cargoes into the tumor cell cytoplasm, the tumor-selective delivery of nucleic acids into the cell cytoplasm could also be improved, e.g., as guide RNA for co-delivered Cas nucleases or as siRNA to interfere with transcription. Currently, it is unclear what cytoplasmic concentrations of delivered cargo molecules can be achieved using internalizing antibodies. A better understanding of the uptake and endosomal escape mechanism may be required to optimize cytoplasmic delivery through rational design and/or high-throughput screening methods.

## Data Availability

The original contributions presented in the study are included in the article/Supplementary Material. Further inquiries can be directed to the corresponding author.

## Supplementary Materials

The following supporting information can be downloaded at: https://www.mdpi.com/article/10.3390/antib13020037/s1, Figure S1: On-cell binding assay of CPAb and CPAb (118S–121S) using HSPG-positive or -negative HeLa cells. Figure S2: On-cell binding assay of CPAb and HerT4 using SKBR-3 cell. Figure S3: Flow cytometry data of antibody-TAMRA conjugates. Figure S4: SDS-gel and flow cytometry data of antibody–GFP conjugates Figure S5: SDS-PAGE of PE_cat_-coupled antibodies, in comparison to uncoupled antibodies. Figure S6: Analytical RP-HPLC chromatogram of HiBiT peptide. Figure S7: Analytical MS(ESI) of HiBiT peptide. Figure S8: Workflow to find paratope-blocking anti-CPAb scFvs. Figure S9: ELISA-based titer determination of chicken immunized with CPAb scFv. Figure S10: Screening campaign of chicken-derived scFv immune library for the isolation of CPAb binders via FACS. Figure S11: Single clone analysis of chicken-derived anti-CPAb scFvs. Figure S12: Alignment of chicken-derived CPAb binders. Figure S13: On-cell binding assay of S4-CPAb (masked and unmasked) using HSPG-positive or -negative HeLa cells (heparinase treated). Figure S14: Aggregation behavior of paratope-blocking scFv-CPAb fusion variants. Figure S15: SDS-Gel of masked antibody-PE_cat_ conjugates, in comparison to MMP-9 cleaved variants. Figure S16: NanoBiT assay analyzing S4-CPAb variants, in comparison with CPAb and CPAb (118S–121S) in HeLa 11ht LgBiT cells. Figure S17: PE_cat_-mediated proliferation assay with masked antibody-PE_cat_ conjugates using HeLa cells. Table S1: Amino acid sequences in one-letter code of the CPAb heavy and light chain and of TEV-cleaved (G_4_S)_2_PE_cat_ [85,86,87,88].
