# Supplementary material for: A Conditionally Activated Cytosol-Penetrating Antibody for TME-Dependent Intracellular Cargo Delivery
Source: Antibodies (Basel). 2024 May 2;13(2):37. doi: 10.3390/antib13020037 (PMC11130931; doi:10.3390/antib13020037)
Supplement: Supplementary file 1 [file antibodies-13-00037-s001.zip › antibodies-2951465-supplementary.pdf]

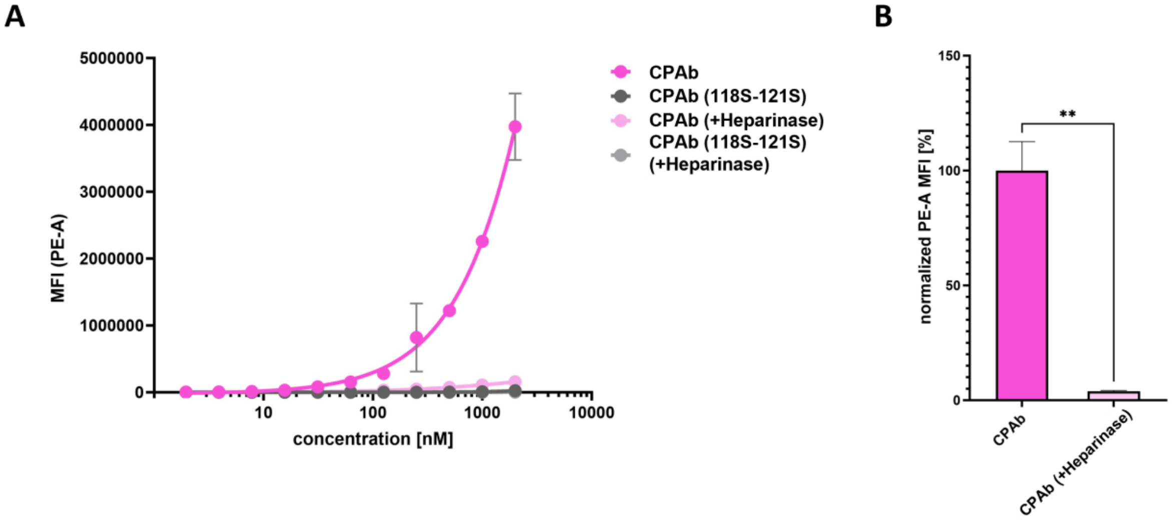

Figure S1. On-cell binding assay of CPAb and CPAb (118S–121S) using HSPG positive or negative HeLa cells. HSPG negative cells were prepared by treatment with Heparinase I and III mix (Sigma Aldrich) prior to the binding assay. Cells were stained with antibody concentrations ranging from 2 nM to 2000 nM. As secondary antibody, a Goat anti-Human IgG Fc, eBioscience™ PE (Invitrogen) was used. Resulting data were derived from experimental duplicates and are shown as mean values and error bars representing standard deviation using GraphPad Prism 10.1.0 (316). CPAb data set is displayed as (**A**) binding curve in comparison to CPAb (118S–121S) and as (**B**) bar graph comparing the normalized MFI of CPAb on positive and negative cells at the highest concentration with HSPG positive cells set as 100%. An unpaired two-tailed t-test (with P value style GP: 0.1234 (ns), 0.0332 (*), 0.0021 (**)) was used to display the significance level (with definition of statistical significance: $P < 0.05$).

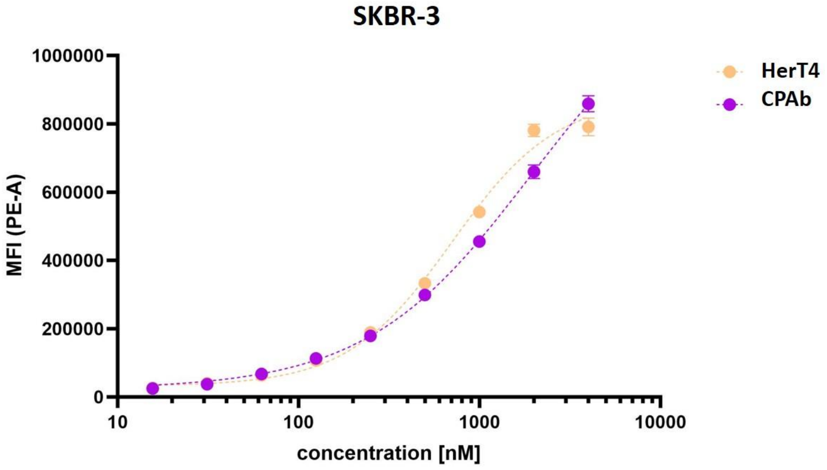

Figure S2. On-cell binding assay of CPAb and HerT4 using SKBR-3 cells. Cells were treated with concentrations ranging from 15 nM to 4000 nM. As secondary antibody, a Goat anti-Human IgG Fc, eBioscienceTM PE (Invitrogen) was used. Resulting data were derived from experimental duplicates and were shown as mean values and error bars representing standard deviation. EC50 values were calculated from variable slope four-parameter fitting using GraphPad Prism 10.1.0 (316).

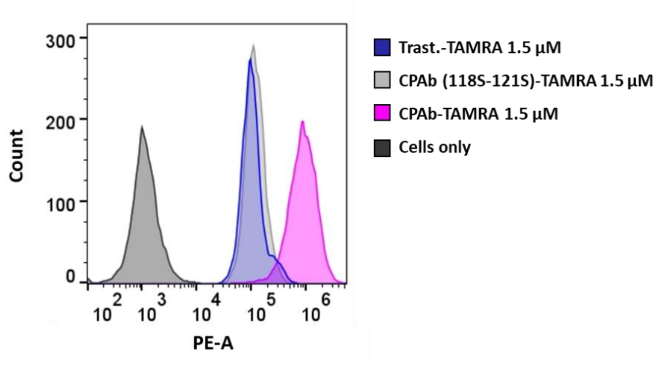

Figure S3. Flow cytometry data of antibody-TAMRA conjugates. Quantitative FACS analysis using 1.5 µM per TAMRA-coupled antibody (trastuzumab, CPAb, or CPAb (118S–121S)) after 8 h incubation time on HeLa cells corresponding to the CLSM images in Figure 3. 10,000 events per sample were recorded.

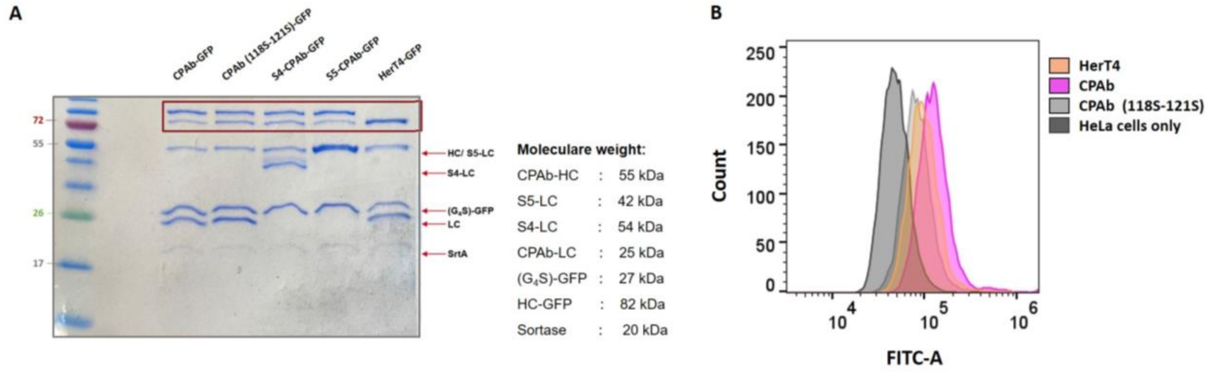

Figure S4. SDS-gel and flow cytometry data of antibody-eGFP conjugates. (**A**) 2 µg of CPAb-eGFP, CPAb (118S–121S)-eGFP, S4-CPAb-eGFP, S5-CPAb-eGFP, and HerT4-eGFP were loaded on the reducing SDS-gel. Color Prestained Protein Standard, Broad Range (10–250 kDa) (NEB) was utilized as marker. The red frame highlights the eGFP-coupled heavy chain. (**B**) Quantitative FACS analysis of the GFP-coupled antibodies after 8 h incubation time on HeLa cells corresponding to the CLSM images in Figure 4. 10,000 events per sample were recorded.

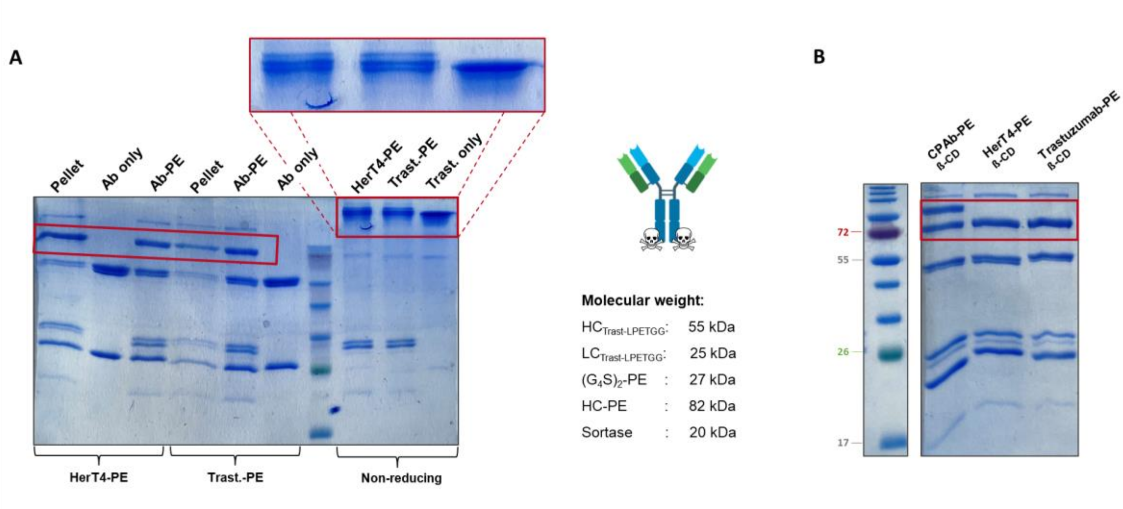

Figure S5. SDS-PAGE of PE_{cat}-coupled antibodies in comparison to uncoupled antibodies. Color Prestained Protein Standard, Broad Range (10–250 kDa) (NEB) was utilized as marker. The red frame highlights the PE_{cat}-coupled heavy chain. (**A**) 2 µg of each antibody was loaded (as pellet, antibody only, and antibody-PE_{cat} conjugate, respectively) to the acrylamide gel. The antibodies were loaded using reducing (left) and non-reducing (right) loading buffer. Zoomed image of the non-reducing samples for better visualization of band separation. (**B**) Reducing SDS-PAGE of PE_{cat}-coupled antibodies with supplemented ß-cyclodextrin to prevent protein aggregation.

CGSSG-HiBiT peptide

Sequence: CGSSG-VSGWRLFKKIS-NH_2

Chemical formula:$C_{76}H_{122}N_{22}O_{21}S$

Molecular weight: 1710.98 g/mol

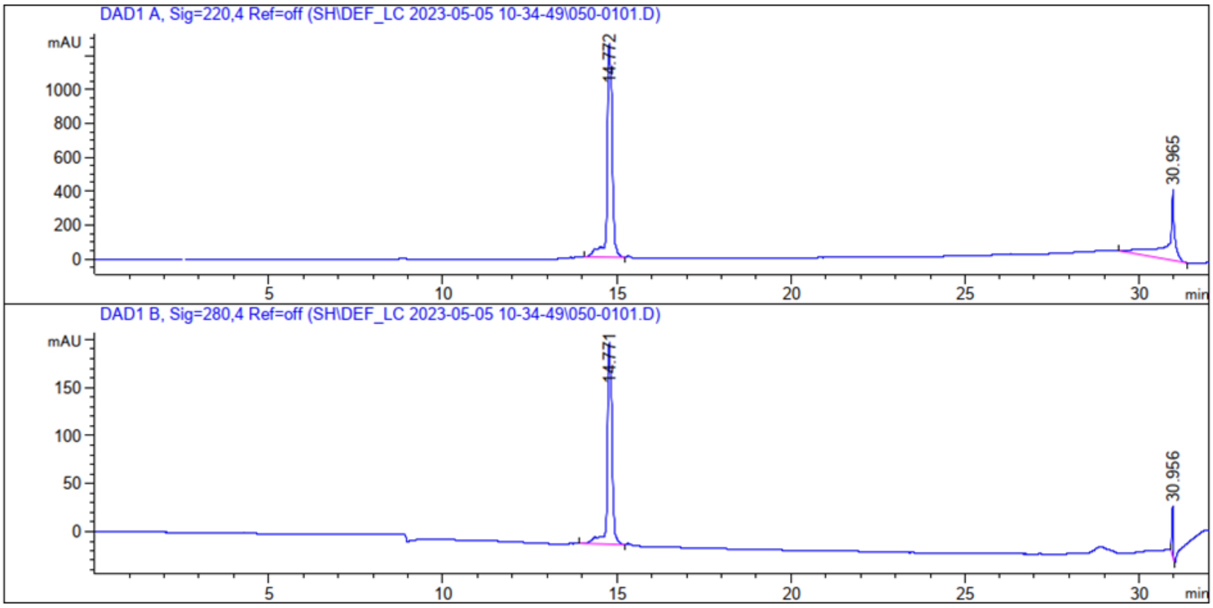

Figure S6. Analytical RP-HPLC chromatogram, 0 to 80% B, 20 min gradient at 0.6 mL/min, 220 nm (top) and 280 (nm) bottom of C-GSSG-HiBiT tR = 14.772 min.

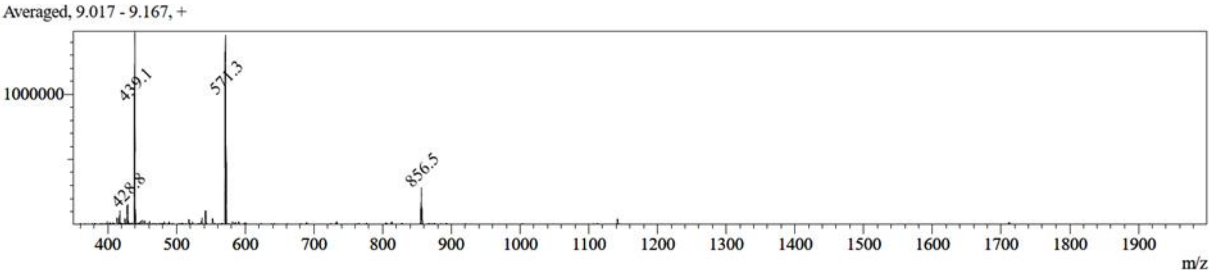

Figure S7. MS(ESI) calculated: [M+H]+ = 1712.0; [M+2H]2+ = 856.5; [M+3H]3+ = 571.3; [M+4H]4+ = 428.8; observed: [M+2H]2+ = 856.5; [M+3H]3+ = 571.3; [M+4H]4+ = 428.8.

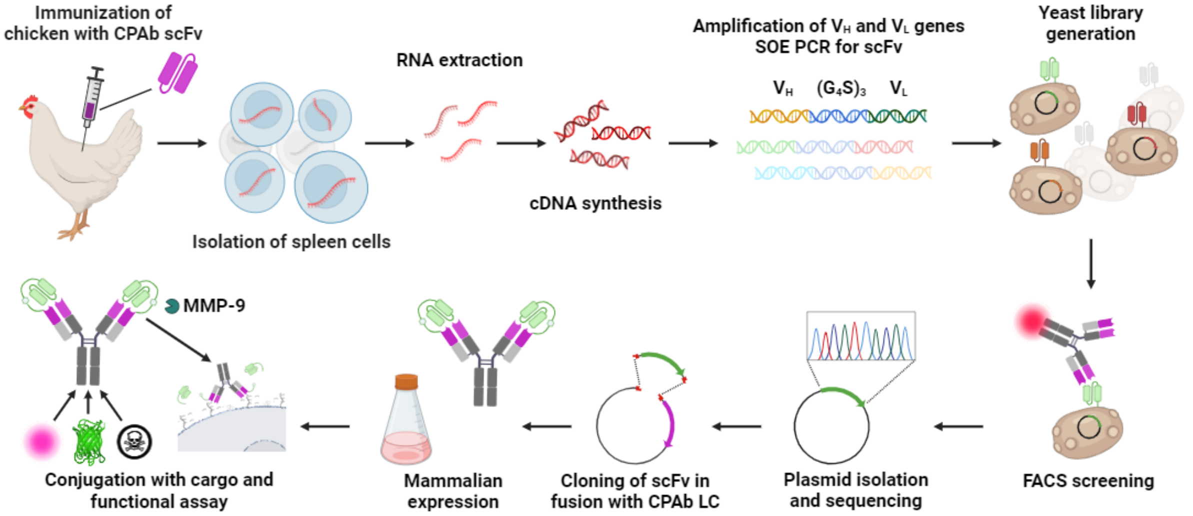

Figure S8. Workflow to find paratope blocking anti-CPAb scFvs. Chicken immunized with recombinantly produced CPAb scFv. RNA extracted from isolated spleen cells for cDNA synthesis. Amplification and assembly of V_H and V_L genes via PCR. Transformation with yeast surface display plasmid into *Saccharomyces cerevisiae*. Library screening for CPAb binders via fluorescence-activated cell sorting (FACS) with fluorescently labeled CPAb. Single cell analysis followed by plasmid isolation and sequencing. Genetic fusion of scFv sequence to N-terminus of CPAb with MMP-9 cleavable linker in mammalian expression vector. Production in Expi293F™ HEK cells and purification via protein A affinity chromatography. Enzyme mediated coupling of fluorophores (TAMRA, eGFP) or toxin (PE_{cat}) for read-out in functional assays. Blocking and MMP-9 mediated restoration of CPAb internalization evaluated via fluorescence microscopy or killing assay, respectively. Figure was created using BioRender.com.

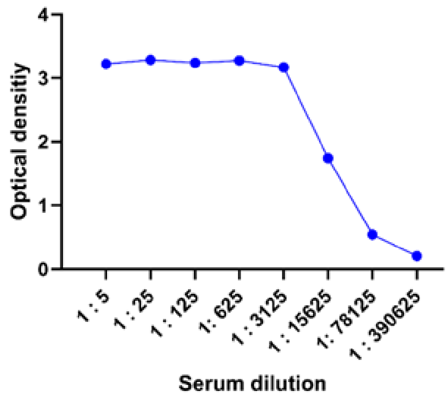

Figure S9. ELISA-based titer determination of one chicken immunized with CPAb scFv. Serial dilution (1:5) of CPAb scFv tested with serum collected after the third booster. Immunization and ELISA performed by Davids Biotech GmbH (Regensburg, Germany).

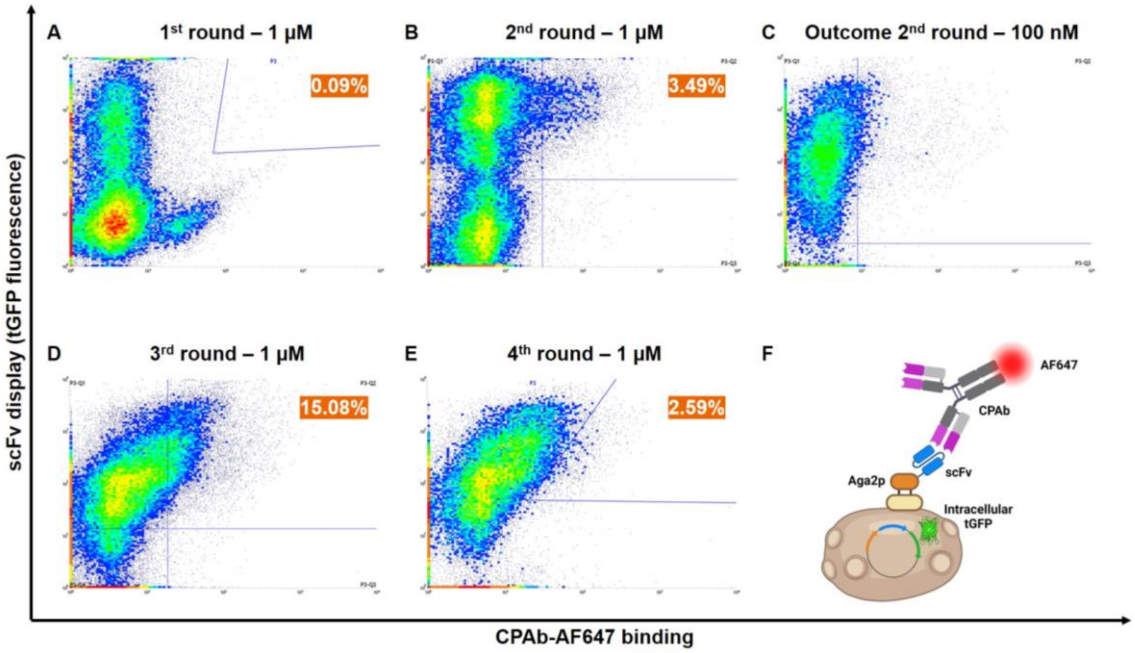

Figure S10. Screening campaign of chicken-derived scFv immune library for the isolation of CPAb binders via FACS. Yeast surface display library of chicken derived anti-CPAb scFvs utilizing tGFP fluorescence as control for induction and surface presentation (y-axis). All sorting rounds were performed with 1 µM Alexa Fluor 647 (AF647) labeled CPAb (x-axis). (**A**) Round 1: 2×10^8 total events screened and 7×10^4 events (0.09%) sorted. (**B**) Round 2: 2×10^6 total events screened and 5×10^4 events (3.49%) sorted. (**C**) Outcome of 2nd sorting round stained with 100 nM target showing presence of CPAb binders with potentially higher affinity. (**D**) Round 3: 3.510^5 total events screened and 5×10^4 events (15.08%) sorted. (**E**) Round 4: 3.2×10^5 total events screened and 8000 events (2.59%) sorted for single clone analysis. 50,000 events per sample recorded and contour plot overlays generated with BD Influx FACS software 1.0.0.650. (**F**) Concept: Yeast surface display of scFv C-terminally fused to Aga2p and indirect surface presentation detection via tGFP expression after ribosomal skipping. Concept created using BioRender.com and adapted from [1,2].

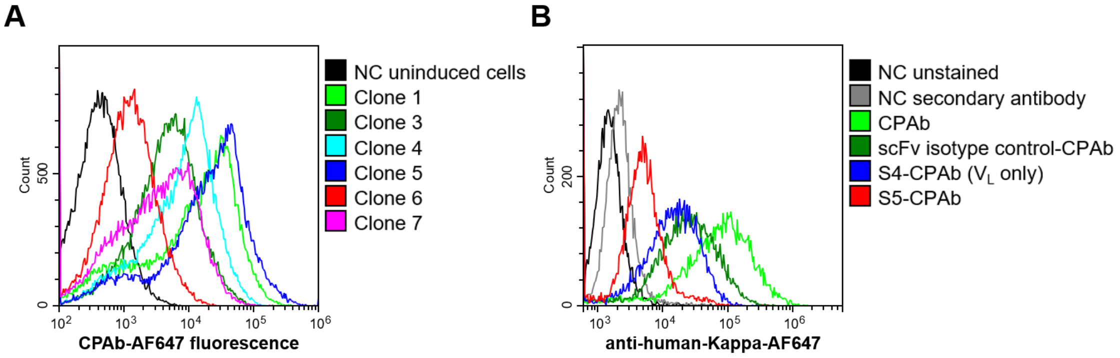

Figure S11. Single clone analysis of chicken-derived anti-CPAb scFvs. (**A**) Six out of ten yeast single clones after the 4th soring round stained with 1 µM CPAb-AF647. (**B**) HeLa wildtype cells incubated with 100 nM scFv N-terminal light chain fusion variants of CPAb for 30 min on ice. Comparison with a non-binding scFv isotype control (ahCG). Detection via Goat F(ab')2 Anti-Human Kappa-AF647 (BIOZOL) as secondary antibody. 10,000 events per sample recorded and histogram overlays generated with CytExpert 2.4.0.28 (Beckman Coulter).

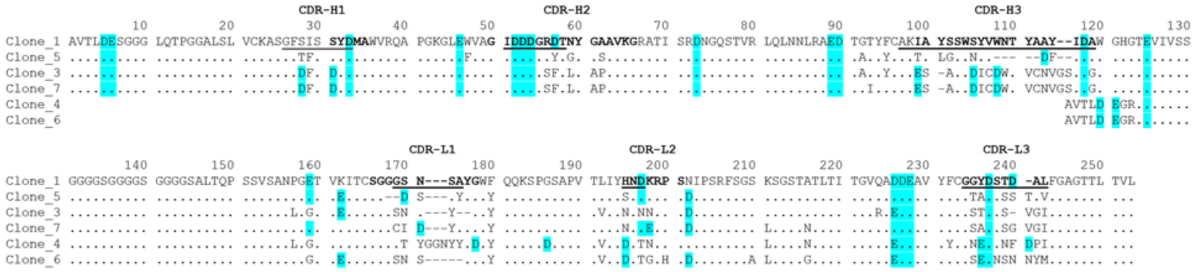

Figure S12. Alignment of chicken-derived CPAb binders. CDR sequences exemplarily annotated for clone 1 according to Kabat (in bold) and IMGT (underlined) numbering [3–5]. Negative residues highlighted in cyan. Multiple sequence alignment performed with MultAlin [6].

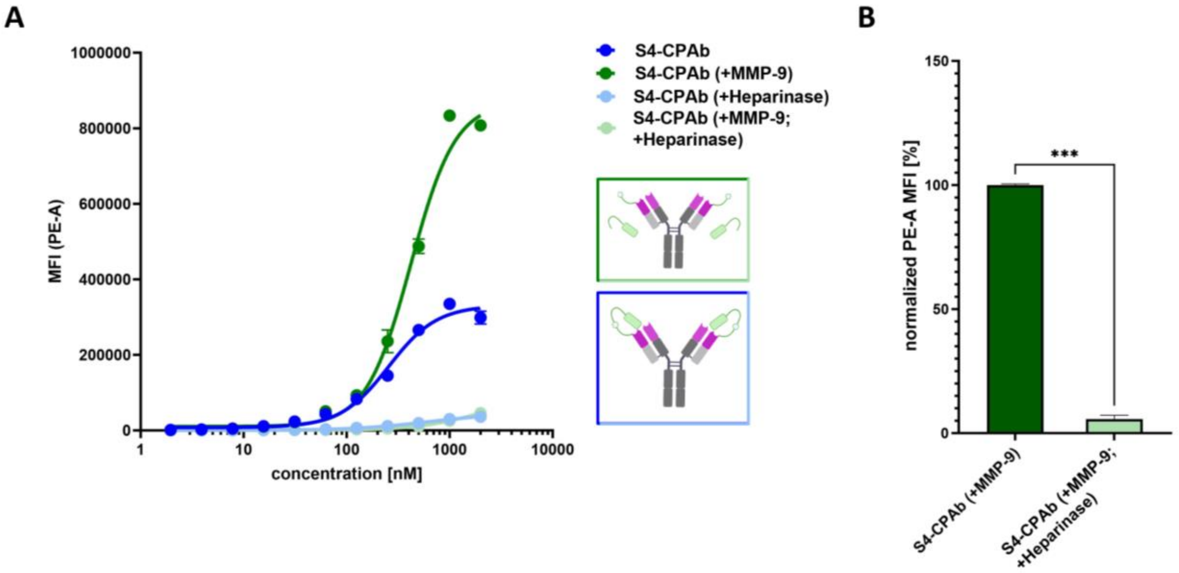

Figure S13. On-cell binding assay of S4-CPAb (masked and unmasked) using HSPG positive or negative HeLa cells. HSPG negative cells were prepared by treatment with Heparinase I and III mix (Sigma Aldrich) prior to the binding assay. Cells were stained with antibody concentrations ranging from 2 nM to 2000 nM. Goat anti-Human IgG Fc, eBioscience™ PE (Invitrogen) was used secondary antibody. Resulting data were derived from experimental duplicates and were shown as mean values and error bars representing standard deviation. The data set of unmasked S4-CPAb (after MMP-9 cleavage) is displayed in (**A**) as binding curve in comparison to masked antibody, and in (**B**) as bar graph comparing the unmasked S4-CPAb on HSPG positive and negative cells at the highest utilized concentration (2 μM) normalized to the sample with HSPG positive cells. An unpaired two-tailed t-test (with P value style GP: 0.1234 (ns), 0.0332 (*), 0.0021 (**), and 0.0002 (***)) was utilized to display the significance level (with definition of statistical significance: $P < 0.05$).

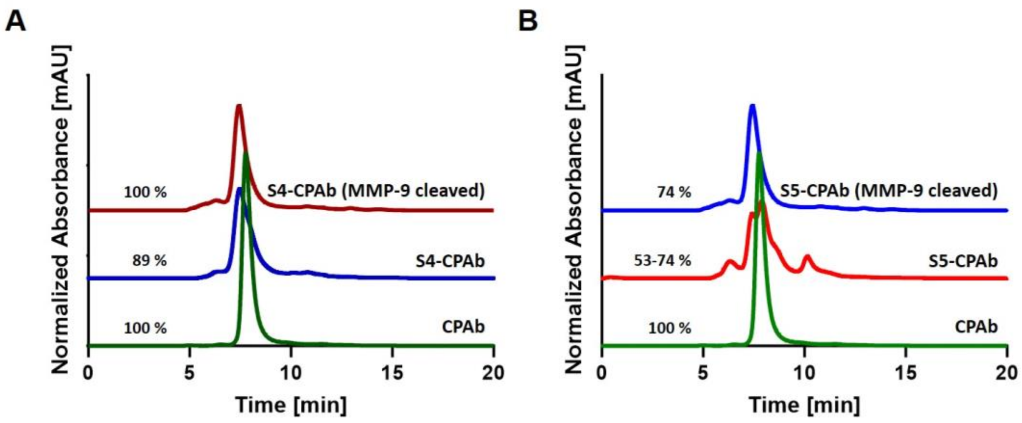

Figure S14. Aggregation behavior of paratope blocking scFv-CPAb fusion variants. (**A**) S4 V_L only or (**B**) S5 scFv were fused N-terminally to the light chain of CPAb via MMP-9 cleavable linker by Geiger et al., 2020 [7]. Expression in Expi293F™ HEK cells, purification via Protein A, dialyzed against TN buffer (50 mM Tris, 150 mM NaCl, pH 7.4), and analyzed via size exclusion chromatography. Comparison with MMP-9 cleaved sample and unmodified CPAb. Respective monomer content depicted in percent. Visualized with GraphPad Prism 10.1.0 (316).

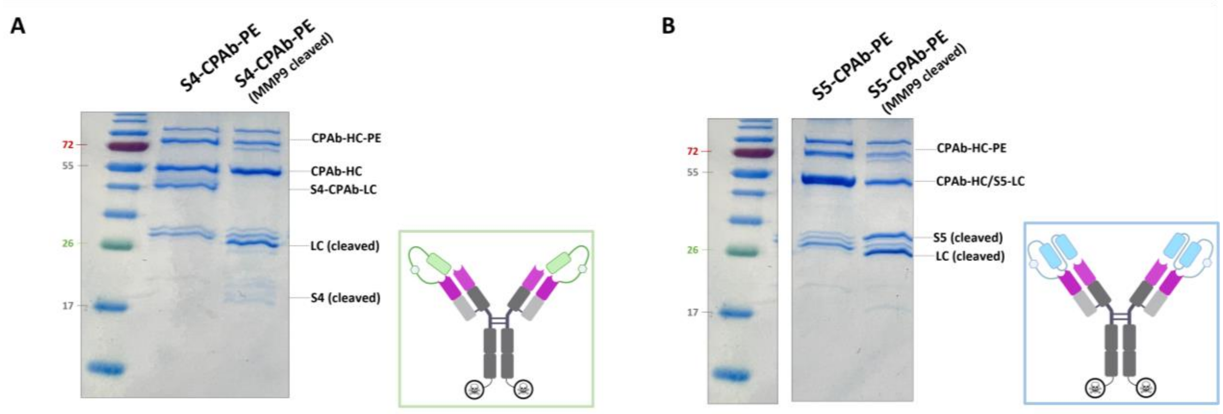

Figure S15. SDS-Gel of masked antibody-PE_{cat} conjugates in comparison to MMP-9 cleaved variants. Color Prestained Protein Standard, Broad Range (10–250 kDa) (NEB) was utilized as marker. 2 µg of each antibody was loaded to the SDS-gel. (**A**) S4- and (**B**) S5-CPAb variants show coupled heavy chains at approximately 82 kDa. Schematic illustration of the respective masked antibody is shown to the right of each SDS-gel.

A

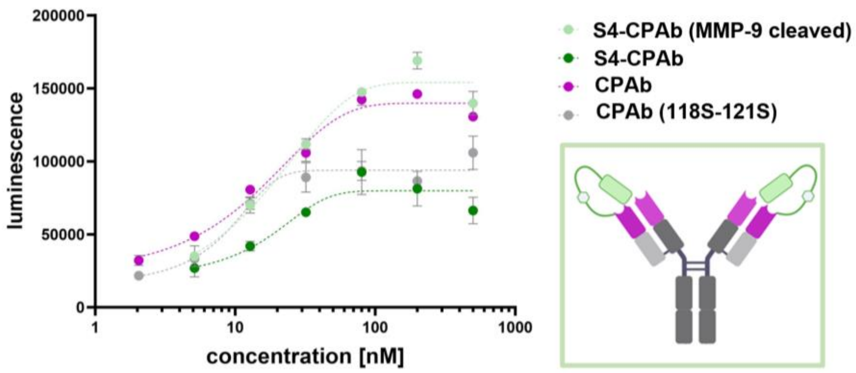

B

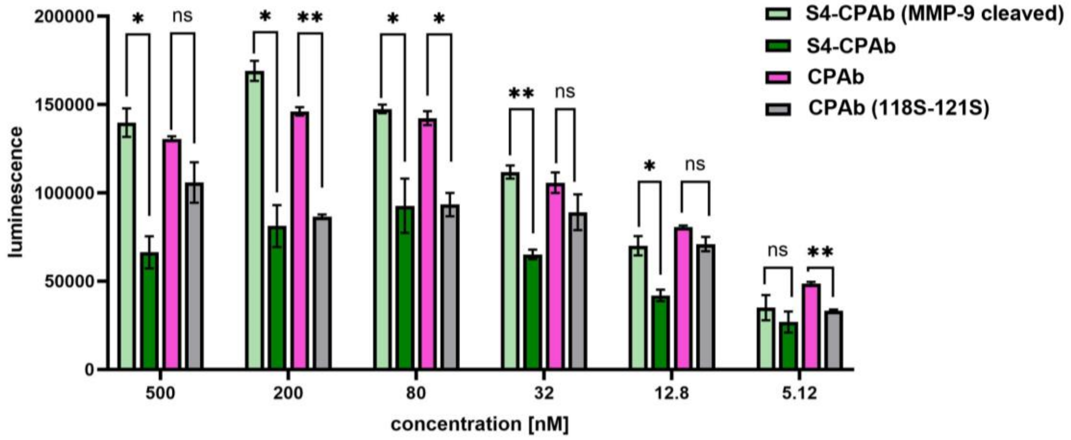

Figure S16. NanoBiT assay analyzing S4-CPAb variants in comparison with CPAb and CPAb (118S–121S) in HeLa 11ht LgBiT cells. Cells were treated for 24 h with different concentrations (5.12 nM–500 nM) of S4-CPAb-HiBiT$_2$, S4-CPAb-HiBiT$_2$ (MMP-9 cleaved), CPAb-HiBiT$_2$, or CPAb(118S–121S)-HiBiT$_2$. The results from experimental duplicates were shown as mean values with error bars representing the standard deviation. (**A**) shows XY plot of the antibody-HiBiT$_2$ concentrations against the respective luminescence intensity. The resulting curves were fitted using a variable slope four-parameter fit. Graph (**B**) depicts the results of (**A**) as bar graph with calculated significances of the S4-variants and CPAb variants, respectively. An unpaired two-tailed t-test (with P value style GP: 0.1234 (ns), 0.0332 (*), and 0.0021 (**)) was used to display the significance level (with definition of statistical significance: $P < 0.05$) using GraphPad Prism 10.1.0 (316).

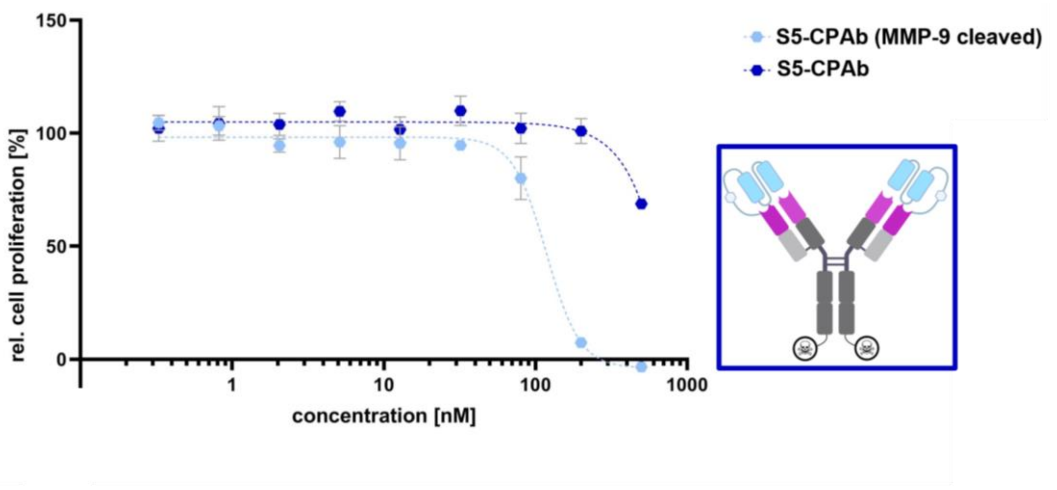

Figure S17. PE_{cat}-mediated proliferation assay with masked antibody-PE_{cat} conjugates using HeLa cells. The S5-CPAb-PE_{cat} conjugates, uncleaved and MMP-9 cleaved, were added to the cells for 72 h in a dilution series ranging from 0.2 to 500 nM. The EC_{50} value of S5-CPAb-PEcat (156 nm) was determined from variable slope four-parameter fitting using GraphPad Prism 10.1.0 (316).

Table S1. Amino acid sequences in one-letter code of the CPAb heavy and light chain and of TEV cleaved $(G_4S)_2$-PE_{cat}. Important AA sequences of the CPAb were highlighted: CDRs are highlighted in bold, the HSPG binding motif is underlined, and the presumed endosomal escaping motif is marked in red.

CPAb-HC	METDTLLLWVLLLWVPGSTGQVQLQESGGGLVQPGGSLRLSCSAS**GFDFSTYGMH**WVRQAPGKGLVWVS**RINSDGSSTSYADSVKG**RFTISRDNAKNTLYLQMNSLRAEDTAVYYC**ARRRRHFDYW**GQGIMVTVSSASTKGPSVFPLAPSSKSTSGGTAALGCLVKDYFPEPVTVSWNSGALTSGVHTFPAVLQSSGLYSLSSVVTVPSSSLGTQTYICNVNHKPSNTKVDKRVEPKSCDKTHTCPPCPAPELLGGPSVFLFPPKPKDTLMISRTPEVTCVVVDVSHEDPEVKFNWYVDGVEVHNAKTKPREEQYNSTYRVVSVLTVLHQDWLNGKEYKCKVSNKALPAPIEKTISKAKGQPREPQVYTLPPSREEMTKNQVSLTCLVKGFYPSDIAVEWESNGQPENNYKTTPPVLDSDGSFFLYSKLTVDKSRWQQGNVFSCSVMHEALHNHYTQKSLSLSPGK
CPAb-LC	METDTLLLWVLLLWVPGSTGAIQLTQAPSSLSASVGDRVTITC**RPSQTISNSLN**WYQQKPGKAPKLLIF**GASSLQG**GAPSRFSGSGSGTDFTLTISSLQPEDFATYYC**QQSFTTPQT**FGQGTKVEIKRTVAAPSVFIFPPSDEQLKSGTASVVCLLNNFYPREAKVQWKVDNALQSGNSQESVTEQDSKDSTYSLSSTLTLSKADYEKHKVYACEVTHQGLSSPVTKSFNRGEC
$(G_4S)_2$-PE_{cat}	GGGGGSGGGGSPTGAEFLGDGGDISFSTRGTQNWTVERLLQAHRQLEERGYVFVGYHGTFLEAAQSIVFGGVRARSQDLDAIWRGFYIAGDPALAYGYAQDQEPDARGRIRNGALLRVYVPRSSLPGFYRTSLTLAAPEAAGEVERLIGHPLPLRLDAITGPEEEGGRLETILGWPLAERTVVIPSAIPTDPRNVGGDLDPSSIPDKEQAISALPDYASQPGKPPHHHHHHGSWSHPQFEKGGGSGGGSGGSSAWSHPQFEK

References

1. Bogen, J.P.; Grzeschik, J.; Krah, S.; Zielonka, S.; Kolmar, H. Rapid Generation of Chicken Immune Libraries for Yeast Surface Display. *Methods Mol. Biol.* **2020**, *2070*, 289–302.
2. Hinz, S.C.; Elter, A.; Grzeschik, J.; Habermann, J.; Becker, B.; Kolmar, H. Simplifying the Detection of Surface Presentation Levels in Yeast Surface Display by Intracellular tGFP Expression. *Methods Mol. Biol.* **2020**, *2070*, 211–222.

3. Dunbar, J.; Deane, C. M. ANARCI: Antigen receptor numbering and receptor classification. *Bioinformatics* **2016**, *32*, 298–300.
4. Ehrenmann, F.; Kaas, Q.; Lefranc, M.-P. IMGT/3Dstructure-DB and IMGT/DomainGapAlign: A database and a tool for immunoglobulins or antibodies, T cell receptors, MHC, IgSF and MhcSF. *Nucleic Acids Res*. **2010**, *38*, D301-307.
5. Ehrenmann, F.; Lefranc, M.-P. IMGT/DomainGapAlign: IMGT standardized analysis of amino acid sequences of variable, constant, and groove domains (IG, TR, MH, IgSF, MhSF). *Cold Spring Harb. Protoc*. **2011**, 737–749.
6. Corpet, F. Multiple sequence alignment with hierarchical clustering. *Nucleic Acids Res*. **1988**, *16*, 10881–10890
7. Geiger, M.; Stubenrauch, K.-G.; Sam, J.; Richter, W.F.; Jordan, G.; Eckmann, J.; Hage, C.; Nicolini, V.; Freimoser-Grundschober, A.; Ritter, M.; et al. Protease-activation using anti-idiotypic masks enables tumor specificity of a folate receptor 1-T cell bispecific antibody. *Nat. Commun.* **2020**, *11*, 3196.
